# Genome-wide association study and transcriptome analysis dissect the genetic control of silique length in *Brassica napus* L.

**DOI:** 10.1186/s13068-021-02064-z

**Published:** 2021-11-07

**Authors:** Jia Wang, Yueling Fan, Lin Mao, Cunmin Qu, Kun Lu, Jiana Li, Liezhao Liu

**Affiliations:** 1 College of Agronomy and Biotechnology, Southwest University, Beibei, Chongqing, China; 2 Academy of Agricultural Sciences, Southwest University, Beibei, Chongqing, China

**Keywords:** *Brassica napus*, Silique length, QTL, GWAS, WGCNA, Meta-GWAS

## Abstract

**Background:**

Rapeseed is the third-largest oilseed crop after soybeans and palm that produces vegetable oil for human consumption and biofuel for industrial production. Silique length (SL) is an important trait that is strongly related to seed yield in rapeseed. Although many studies related to SL have been reported in rapeseed, only a few candidate genes have been found and cloned, and the genetic mechanisms regulating SL in rapeseed remain unclear. Here, we dissected the genetic basis of SL by genome-wide association studies (GWAS) combined with transcriptome analysis.

**Results:**

We identified quantitative trait locus (QTL) for SL using a recombinant inbred line (RIL) population and two independent GWAS populations. Major QTLs on chromosomes A07, A09, and C08 were stably detected in all environments from all populations. Several candidate genes related to starch and sucrose metabolism, plant hormone signal transmission and phenylpropanoid biosynthesis were detected in the main QTL intervals, such as *BnaA9.CP12-2*, *BnaA9.NST2*, *BnaA7.MYB63*, and *BnaA7.ARF17*. In addition, the results of RNA-seq and weighted gene co-expression network analysis (WGCNA) showed that starch and sucrose metabolism, photosynthesis, and secondary cell wall biosynthesis play an important role in the development of siliques.

**Conclusions:**

We propose that photosynthesis, sucrose and starch metabolism, plant hormones, and lignin content play important roles in the development of rapeseed siliques.

**Supplementary Information:**

The online version contains supplementary material available at 10.1186/s13068-021-02064-z.

## Introduction

*Brassica napus* L., an amphidiploid species formed by natural hybridization of *Brassica rapa* and *Brassica oleracea*, is an important oilseed crop with strong adaptability, wide use and high economic value. As the important oilseed crop in the world, *Brassica napus* (*B. napus*) is cultivated worldwide and is increasingly used for animal feed, vegetable oil and biodiesel [1]. Therefore, increasing rapeseed yield is one of the important goals of *B. napus* breeding and cultivation. Silique length (SL) is one of the yield-determining traits in *B. napus* [2, 3]. Silique plays an important role in the yield formation of *B. napus*. It is not only a sink organ for absorbing and accumulating photosynthetic products produced by leaves but also a source organ for seed development [4]. In the late stage of seed development, the functional leaf area of *B. napus* decreases rapidly, and photosynthesis of green silique becomes the main source of nutrition for seed development [5]. Long siliques generally have a large photosynthetic area to potentially produce more energy; they consume large amounts of energy for their development. Therefore, a proper silique length is needed to balance the processes of producing, transferring, and consuming energy in silique to optimize seed number and size [6]. In general, long siliques produce more or larger seeds than short siliques. Under the same planting density, silique number per plant, seed number per silique, and seed weight are the three direct components that determine the seed yield per plant [7]. Therefore, it is of great significance to determine the genetic basis of long silique and cultivate long siliques rapeseed varieties to improve the yield of rapeseed.

Most rapeseed agronomic traits including SL are complex quantitative traits controlled by multiple genes and are influenced by the environment. Genome-wide association analysis (GWAS) and quantitative trait locus (QTL) mapping are effective methods for dissecting complex traits. According to incomplete statistics, more than 100 QTLs for SL have been identified by linkage and association mapping, and QTL controlling SL are distributed on almost all chromosomes, although major QTLs have been found mainly on chromosomes A07, A09, C02, C08, and C09 [1, 6, 8–15]. Based on these results, some candidate genes have been reported. Liu et al. [16] successfully cloned the QTL gene *ARF18*, which is a negative regulator that controls both silique length and seed weight in *B. napus*. One 55-aa deletion prevented *ARF18* forming a homodimer, inhibited the activity of downstream auxin genes, and promoted silique elongation by prolonging the length of pericarp cells [16]. *BnaA9.CYP78A9* was cloned in the major QTL region of chromosome A09 by fine mapping. A 3.7-kb insertion of a CACTA-like transposable element (TE) in the regulatory region functioned as an enhancer to stimulate the expression of *BnaA9.CYP78A9* and silique elongation [10]. The molecular regulation of silique development in *B. napus* is largely unknown, although some important candidate genes have been reported and their relationship with SL has been analysed. Therefore, further understanding of the genetic architecture of rapeseed SL is needed.

With the development of high-throughput sequencing technologies and the continuous decrease in high-throughput sequencing costs, GWAS is increasingly widely used in the study of complex quantitative traits. Increasing the GWAS sample size is the most direct and effective way to improve the test efficiency; however, it incurs a huge workload and expensive experimental costs; and the GWAS of a single small population may not accurately capture the genetic variations. In addition, the GWAS results for the same trait are often inconsistent due to different experimental populations, genotyping, and analysis methods. Meta-analysis provides an attractive alternative to address the abovementioned challenges in the GWAS of single populations, and meta-GWAS have been utilized to detect genetic risk loci for various diseases in humans [17, 18]. In plants, using meta-analyses of a genome-wide association study (meta-GWAS), Battenfield et al. [19] identified marker–trait associations, allele effects, candidate genes, and generated selection markers in bread wheat. Another research group integrated genetic information from 73 published studies including 17,556 soybean germplasm resources, and reported 393 unique peaks including 66 candidate genes across important traits, providing confirmation of many previously reported genes [20]. Fikere et al. [21] identified 79 genomic regions (674 SNPs) conferring potential resistance to canola blackleg by meta-analysis GWAS. Su et al. [22] reported that 3589 significant loci for three component traits and three loci for yield were detected by investigating the four traits of two rice hybrid populations in different environments using meta-GWAS.

In this study, we carried out a QTL analysis with a population of 172 F_9_ RILs and GWAS with two independent populations. In addition, we performed WGCNA with transcriptome sequencing of developing pericarp from short and long silique lines. Specifically, we aimed to detect candidate genes associated with SL and to analyse the genetic bases contributing to the difference between short and long siliques. We suggest that selecting SL may be an effective strategy in breeding to improve rapeseed production.

## Results

### Phenotypic evaluation showed wide variation widely

**Fig. 1:**
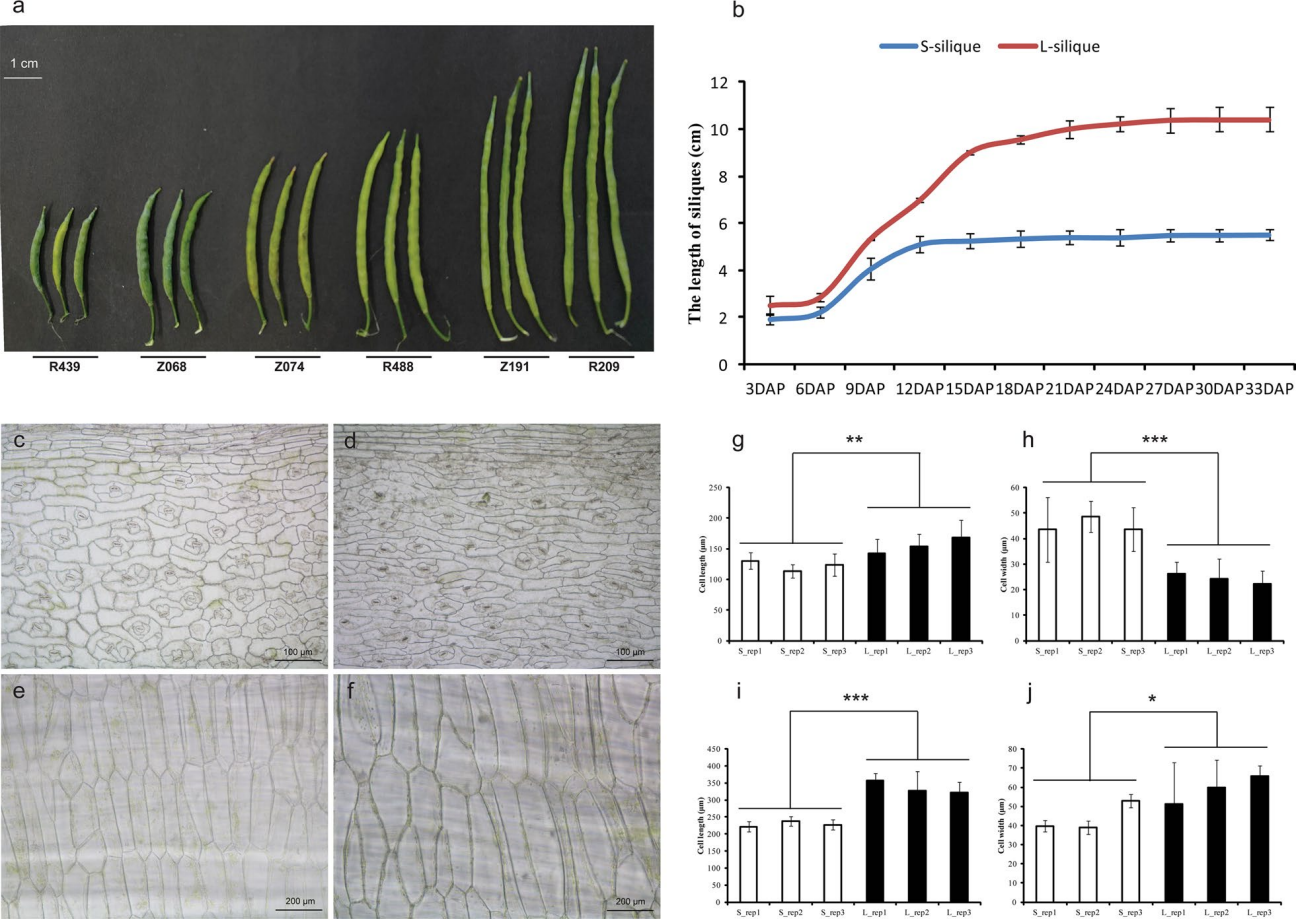
Phenotypic characterization of the short- and long-silique rapeseed. **a** Silique length of different lines. The line of Z number is from RIL population, and the line of R number is from GWAS population. Bar = 1 cm. **b** Dynamic changes of silique lengths of S- and L-siliques in different developmental stages. **c**, **d** Microstructure observation of the outer pericarp of the S-silique materials and L-silique materials, bar = 100 μm. **e**, **f** Microstructure observation of the endocarp of the S-silique materials and L-silique materials, bar = 200 μm. **g** Cell length measurement of outer pericarp. **h** Cell width measurement of outer pericarp. **i** Cell length measurement of endocarp. **j** Cell width measurement of endocarp. Statistically significant differences were revealed using a Student’s *t* test: *p < 0.05; **p < 0.01, ***p < 0.001

We measured the SL of 172 RILs from 2016 to 2019, with five replications performed each year, and measured the SL of 520 accessions during 2015 and 608 lines during 2017, with ten replications performed each year. The results showed that SL differed tremendously among rapeseed lines, with 76.26% broad-sense heritability, ranging from 4.81 to 10.89 cm in the RIL population and ranging from 3.39 to 12.74 cm in the GWAS population; 74.07% of lines were concentrated in the range of 5.00–8.00 cm (Fig. 1a, Additional file 1: Table S1, Additional file 2: Fig. S1a, b). A correlation analysis showed a strong correlation between SL and thousand seed weight (TSW), seed yield per plant (YP), seed number per silique (SN), seed number per plant (SNPP), siliques per the main inflorescence (SMI), and harvest index (HI), and a weak correlation with seed oil content (SOC) (Additional file 3: Fig. S2). The SL variation was further analysed in winter, spring, and semi-winter subgroups. The results indicated that there were differences among the three subgroups, but the differences were not significant. The SL of semi-winter cultivars is 6.07 ± 1.23 cm, while 5.51 ± 1.45 cm and 5.31 ± 1.12 cm in winter and spring accessions, respectively (Additional file 1: Table S2).

We measured the growth rates of siliques using two extreme lines Z068 (5.45 ± 0.29 cm) and Z191 (10.28 ± 1.01 cm), from the RIL population as representatives of short and long siliques, respectively. The results showed that there was no significant difference in SL between long and short siliques in the first 3 days after pollination (DAP). At 9 DAP, there was a significant difference in SL between long and short siliques. The length of the short siliques reached the maximum at 18 DAP, but the length of the long siliques did not stop growing until 27 DAP (Fig. 1b). Further histological observation of the inner and outer epidermis of the long and short siliques at 27 DAP showed that the cell length of the long silique on the outer epidermis was significantly longer than that of the short silique, while the cell width of the long silique was significantly smaller than that of the short silique, and both the cell length and the cell width of the long silique on the inner epidermis were significantly larger than those of the short silique (Fig. 1c–j).

### RNA-seq found that plant hormones transportation and synthesis of carbohydrates involved in the development of rapeseed silique

After a stringent quality filtering process, 79.37 Gb of clean RNA-seq reads were obtained from 12 samples, with a Q30 percentage ≥ 94.80% (Additional file 4: Table S2). The obtained 12 samples of clean reads were mapped to the reference genome sequence of *B. napus*, the percentages of mapped reads were similar among the 12 libraries (86.37–90.23%), and 82.71–86.29% of the reads were uniquely mapped (Additional file 5: Table S3). Based on the mapped results, the FPKM of all genes was counted, the log_10_FPKM values of all samples fluctuated slightly (− 2.5 to 2.5) (Additional file 6: Fig. S3a), and the peak values of most genes were between 0 and 1 (Additional file 6: Fig. S3b). The results of the correlation analysis showed that there was a high correlation and good repeatability between biological replicates (Additional file 6: Fig. S3c). After DEG analysis, 21,482 DEGs remained for further analysis (the number of DEGs was the sum of each DEG set) (Additional file 6: Fig. S3d, e, Additional file 7: Table S4).

To explore the metabolic pathways enriched for the DEGs, the related DEGs in 12 samples were subjected to KEGG metabolic pathway enrichment analysis. In “T1 vs. T2”, we found that the DEGs, whether from S-silique or l-silique, were significantly enriched in starch and sucrose metabolism, phenylalanine metabolism, and phenylpropanoid biosynthesis pathways (Additional file 8: Fig. S4). The same enrichment result also appeared in “T1 vs. T3”, though the difference was that the plant hormone signal transduction pathway, which was only enriched in the L-silique in “T1 vs. T2”, was also enriched in the S-silique in “T1 vs. T3”. In “T2 vs. T3”, the DEGs of L-silique were still enriched in plant hormone signal transduction, starch and sucrose metabolism, phenylalanine metabolism, etc. (Additional file 8: Fig. S4). In the T3 stage, the origin of DEGs was significantly different from the T1 and T2 stages, and the genes related to the cutin, suberine, and wax biosynthesis pathways were significantly up-regulated at this stage. Although there were significant differences in DEGs and enrichment pathways among the three stages, the genes related to starch and sucrose metabolism, plant hormone signal transduction, and phenylpropanoid biosynthesis were differentially expressed in different stages of silique development. These results indicated that the genes related to starch and sucrose metabolism, plant hormone signal transduction, and phenylpropanoid biosynthesis may play important roles in silique development.

### Linkage mapping and GWAS co-located several major QTLs related to SL

Using linkage mapping, we identified 95 QTLs associated with SL, with a logarithm of the odds (LOD) value above 3.0. Of these, 95 QTLs with phenotypic variation explained (PVE) ranging from 0.02 to 67.06% were identified on nine chromosomes using 4 years, BLUE, and BLUP data (Additional file 9: Table S5, Additional file 10: Fig. S5). Three major QTL were located on A07, A09, and C08. *qSLA7-2* on A07 was a major locus that was stably detected across all environments and explained 56.24–67.06% of the phenotypic variation. *qSLA9-1* and *qSLA9-3* on A09 were also major loci that were stably detected across all environments but explained only 0.83–5.33% of the phenotypic variation. *qSLC8-2* on C08 was another major locus that was stably detected across all environments and explained 9.91–11.31% of the phenotypic variation. In addition, *qSLA6-1* and *qSLA6-2* on A06 were detected simultaneously in all environments, but they had a low PVE.

**Fig. 2:**
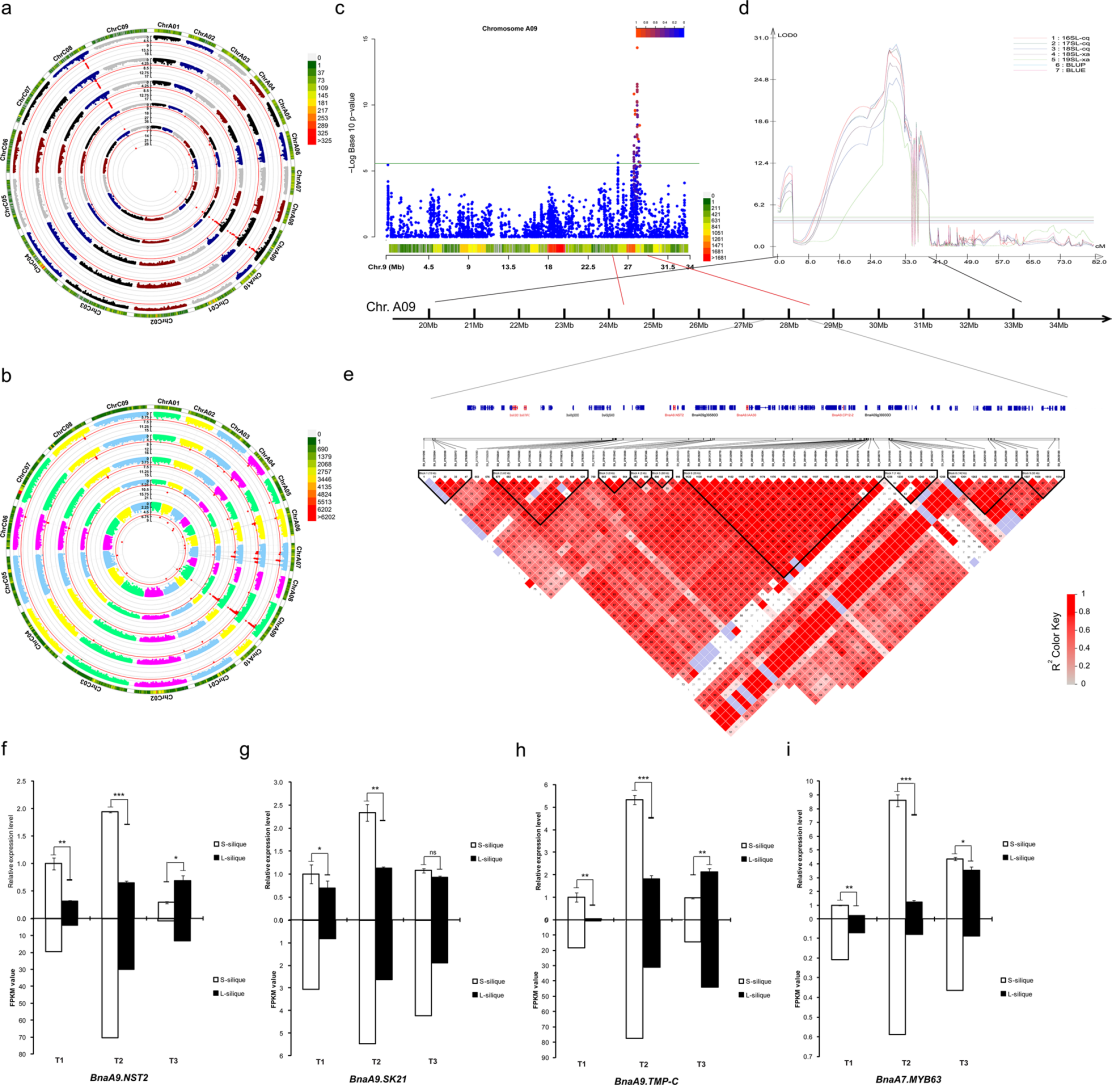
GWAS and QTL co-located of major loci on chromosome A09. **a**, **b** Circular Manhattan plots of the 60 K population and WGR population. From the inner ring to the outer ring are results of the FarmCPU, Blink, CMLM, GLM, and MLM models. **c** Scatterplot of association results from an MLM model analysis of SL on chromosome A09. Negative log10-transformed P values from the GWAS analysis are plotted against the genomic physical position. The green line indicates the threshold level log(1/*N*) = 5.58. **d** Major QTL loci on the chromosome A09 were repeatedly detected in multiple environments by QTL mapping in an RIL population. 16SL-cq, 17SL-cq, and 18SL-cq represent silique length from Chongqing in 2016, 2017, and 2018, respectively; 18SL-xa and 19SL-xa represent silique length from Xi’an in 2018 and 2019, respectively; BLUP and BLUE represent best linear unbiased predictions and best linear unbiased estimates, respectively. **e** Location of the reference genome region on A09 corresponding to the major effective loci and LD block analysis of this region. The red gene ID represents that the gene is an important candidate gene. **f**, **i** FPKM value and qRT–PCR validation of candidate genes in A09. The upper half represents qRT–PCR results, and the normalized levels ST1 were arbitrarily set to 1. The lower part is the FPKM value obtained by RNA-seq. Statistically significant differences were revealed using a Student’s *t* test: ns ≥ 0.05, *p < 0.05, **p < 0.01, ***p < 0.001

Trait–marker associations were performed using the FarmCPU, Blink, CMLM, GLM, and MLM models in two GWAS mapping populations. Similar results were obtained in the two mapping populations with the five models (Fig. 2a, b). To facilitate further analysis, combined with Q–Q diagram, we finally chose the MLM model as the follow-up analysis model (Additional file 11: Fig. S6a, b). In total, we identified 41 SNPs in the 60 K population on chromosomes A06 (1), A07 (6), A09 (15), A10 (1), and C08 (18), whereas 181 SNPs in the whole genome resequencing (WRG) population were identified on chromosomes A01 (9), A03 (9), A04 (11), A06 (10), A07 (52), A08 (5), A09 (54), A10 (4), C01 (1), C04 (1), C05 (5), C06 (1), C07 (4), C08 (9), and C09 (6) using a threshold of 5% after Bonferroni multiple test correction (Additional file 12: Table S6). The SL was associated with three common significant regions located on chromosomes A07, A09, and C08. Among these, 40 SNPs forming a haplotype block on A09 (27.51–28.18 Mb) were located in the interval of known QTL for SL [6].

### Meta-GWAS and polymorphisms in the candidate region were associated with SL

The meta-analysis detected 85 SNPs associated with SL, of which nine SNPs were undetected in all of the single-population GWAS (Additional file 12: Table S6). Fourteen and 31 SNPs identified in the spring and semi-winters subgroups were confirmed by meta-analysis, respectively, but only three SNPs identified in the winter subgroup were confirmed by meta-analysis. On chromosome A09, the confidence interval of a stable major QTL overlapped with the highly associated region detected by GWAS (Fig. 2c, d). Forty SNPs identified in GWAS were confirmed by meta-analysis, and seven SNPs undetected in GWAS were mined by meta-analysis in this interval (Additional file 12: Table S6). 528 gene symbols were found in this region. Among them, 98 were DEGs in RNA-seq, which were listed as candidate genes (Additional file 13: Table S7). LD analysis showed that most peak SNPs were mainly concentrated in block 2 and block 6 (Fig. 2e). The peak SNP (S9_28151819) was involved in a 35-kb LD block (block 6) that encompassed 19 SNP markers, and it was 0.91 kb away from *BnaA9.CP12-2* was related to carbohydrate anabolism (Additional file 13: Table S7). In addition, the gene *BnaA9.NST2* is involved in secondary cell wall biogenesis [23] and the Aux/IAA family member *BnaA9.IAA30* related to auxin signalling was also found in this block. The results of RNA-seq and qRT–PCR showed that the expression of *BnaA9.NST2* in short silique was significantly higher than that in the long silique, especially in the T2 stage (18 DAP) (Fig. 2f).

In block 2, there were two peak SNPs located inside the *BnaA9.SK21* and *BnaA9.TMP-C*, respectively. *BnaA9.SK21* and *BnaA9.TMP-C* had the same expression pattern in T1 and T2, but *BnaA9.TMP-C* had a higher expression level in long siliques in T3 (Fig. 2g, h). Two nonsynonymous SNPs, S9_27782829 and S9_27788376, were present in *BnaA9.SK21* and *BnaA9.TMP-C*, respectively. Further analysis of these two SNPs found that accessions with an AA genotype at S9_27782829 displayed, on average, 18.56% increased silique length compared to the accessions with a TT genotype. The average silique length of AT genotypes was between the AA and TT genotypes. The minor allele (A) was represented in only 17% of the 608 accessions (Additional file 14: Fig. S7a). There was a significant difference between the GG and CC genotypes at the S9_27788376 (*p* < 0.01), and the CC genotype increased silique length (Additional file 14: Fig. S7b).

**Fig. 3:**
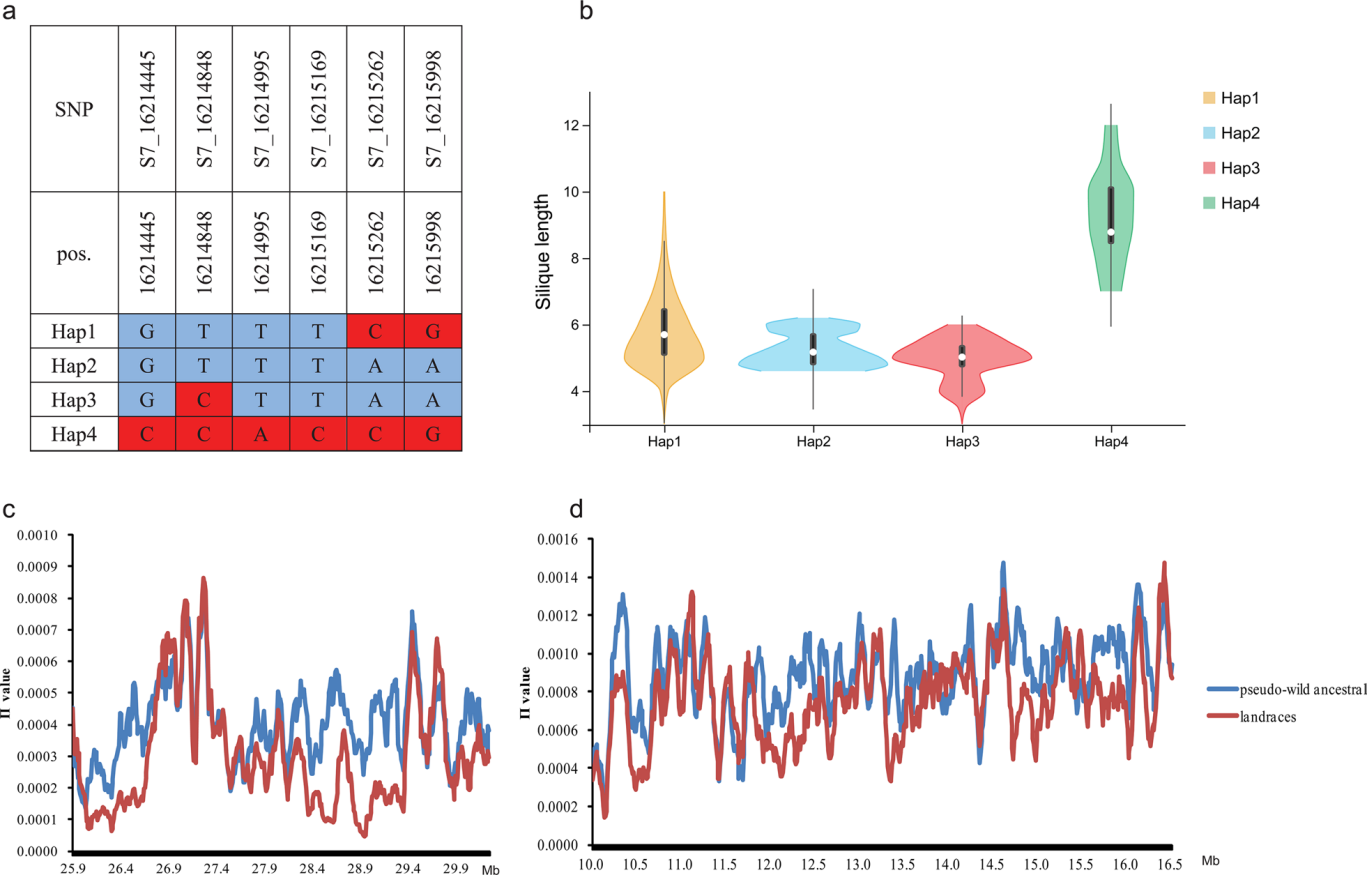
Haplotype analysis and polymorphisms in the candidate region were associated with SL. **a** Haplotypes in 608 accessions (haplotypes with fewer than five accessions were omitted) according to SNP data from WGR population. **b** Violin plots showing the levels of SL from four haplotypes. **c** Genomic diversity of chr A09. **d** Genomic diversity of chr A07. The blue line represents pseudo-wild ancestral and the red line represents landrace rapeseed

Similar results were found on chromosome A07, where a stable major QTL co-located with GWAS. Fourteen SNPs identified in GWAS were confirmed by meta-analysis, and four SNPs undetected in GWAS were mined by meta-analysis in this interval (Additional file 15: Fig. S8). A total of 102 gene symbols were found in this region, of which 46 were DEGs in RNA-seq and listed as candidate genes (Additional file 13: Table S7). *BnaA7.MYB63*, a homologous gene *MYB63*, is a transcriptional regulator specifically activating lignin biosynthetic genes during secondary wall formation in *Arabidopsis thaliana* [24] and was only 0.36 kb away from the significant SNP S7_16015077 with a low expression level during silique development (Fig. 2i). The key lignin biosynthesis gene *BnaA7.CCR2* was also detected in this QTL region and *BnaA7.CCOAMT*, another key gene of lignin biosynthesis, was detected in another highly associated region on A07. Three significant SNPs, S7_16214445, S7_16214995, and S7_16215169, are located inside *BnaA7.ARF17*, which is a negative auxin response factor that inhibits downstream auxin-related genes. Further haplotype analysis was focused on the gene *BnaA7.ARF17*, and four major haplotypes were observed, with low-frequency haplotypes (less than five accessions) being omitted (Fig. 3a). We conducted multiple comparison tests of SL, and the results showed that Hap2 and Hap3 had shorter SL than Hap4 (*P* < 0.05), while Hap1 was an intermediate type (Fig. 3b).

We further analysed the sequence diversity of the major QTL region on Chr A07, A09, and C08 among landraces and pseudo-wild ancestral (European turnip and *B. oleracea* subspecies) genomes based on previously published data [25] (Fig. 3c, d). The landraces had a lower *π* value than pseudo-wild ancestral in the major loci region of A09 (27.50–29.40 Mb). On A07, the highest *π* value of the major QTL region (from approx. 15.80 to 16.4 Mb) was in landraces (1.18 × 10^−3^) and pseudo-wild ancestral (1.36 × 10^−3^). The sequence diversity of the major loci region on chromosome C08 is consistent with A07 and A09 (Additional file 14: Fig. S7c). These results suggested that these major QTLs might be domesticated and selected during the process of rapeseed domestication from wild type to cultivated rapeseed, resulting in a decrease in sequence diversity.

### Co-expression network analysis reveals transcript level differences in photosynthesis and secondary cell wall biosynthesis in long and short siliques

**Fig. 4:**
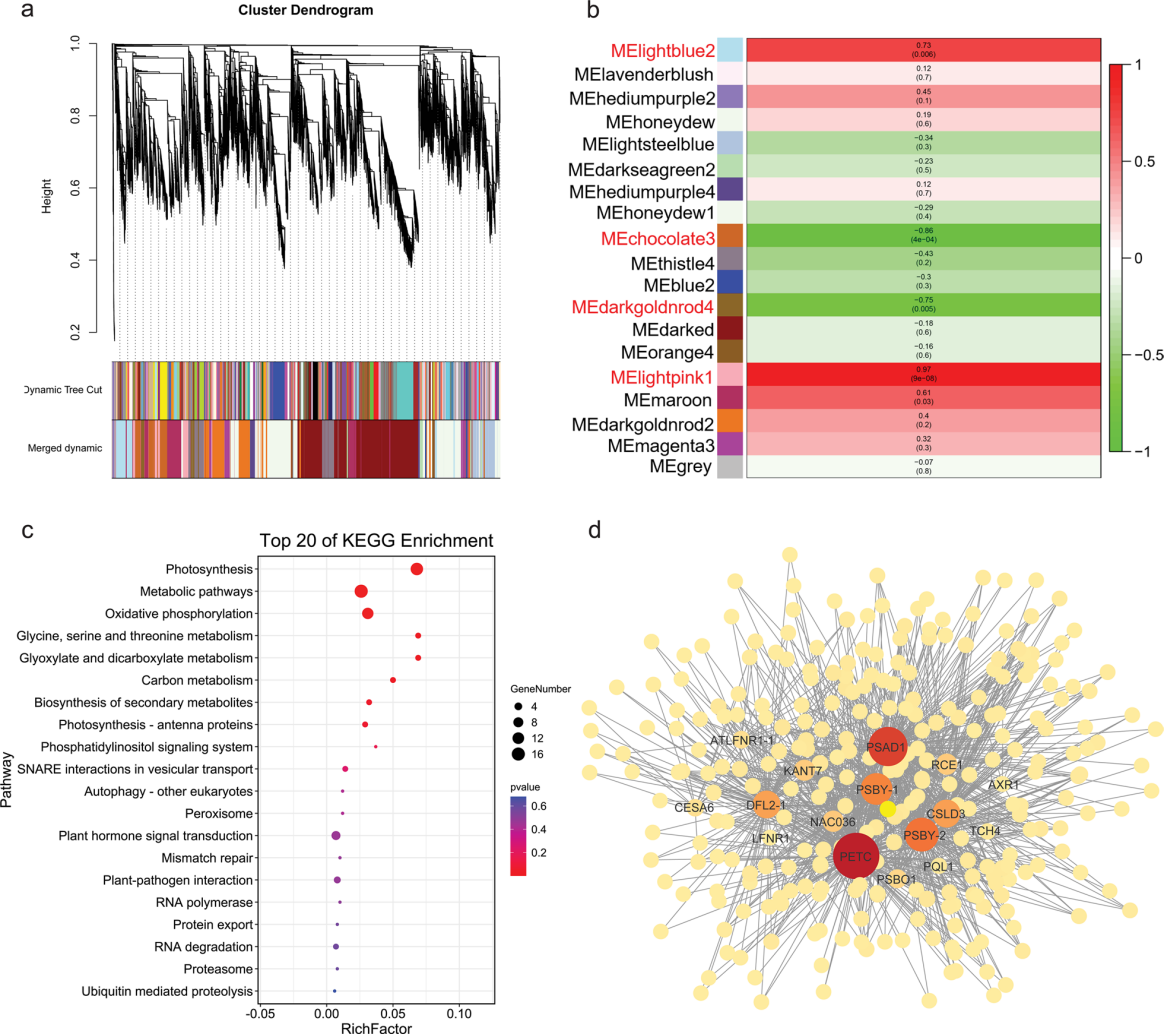
Weighted gene co-expression network analysis. **a** Clustering dendrogram of genes and construction of modules. **b** Phenotype and module correlation analysis heat map. Red indicates that the correlation between module eigengenes and silique length is high. **c** Top 20 KEGG enriched pathways in the set of the lightpink1 module. **d** Gene co-expression network of the lightpink1 module. The node and edge size is proportional to the core

To identify genes related to SL, we performed a weighted gene co-expression network analysis (WGCNA) using the union of non-redundant DEGs and putative candidate genes. After using a dynamic tree cutting algorithm, a total of 18 distinct co-expression modules containing 47 to 1787 genes per module were identified, and 1585 uncorrelated genes were assigned to a grey module, which was ignored in the following study (Fig. 4a). An analysis of the module–trait relationships revealed that the “lightpink1” (*r* = 0.97, *p* = 9e−08), “chocolate3” (*r* = − 0.86, *p* = 4e−04), “darkgoldnrod4” (*r* = − 0.75, *p* = 0.005), and “lightblue2” (*r* = 0.73, *p* = 0.006) module were highly correlated with the SL in the 12 samples (Fig. 4b). According to the heatmap of the top 20 genes with the high eigengene connectivity (KME) value in the four modules, the “lightpink1” module had an expression peak in the T2 stage of long silique development, similar to the long silique development pattern, while genes in the “chocolate3” module were highly expressed in the T3 stage of short silique development (Additional file 16: Fig. S9a, b). The expression level of the top 20 genes in the “darkgoldnrod4” module was the highest in the T1 stage of short silique development, while the expression level of the “lightblue2” module was the highest in the T3 stage of long silique development (Additional file 16: Fig. S9c, d).

A Gene Ontology (GO) enrichment analysis of the “lightpink1” module genes identified ten significantly enriched GO terms, most of which are related to photosynthesis. Interestingly, photosynthesis-related pathways were also enriched in the “lightpink1” module by KEGG pathway enrichment analysis (Fig. 4c, Additional file 17: Table S8). There was no significant enrichment of GO terms in the “chocolate3” module. One and 13 GO terms were significantly enriched in the “darkgoldnrod4” module and “lightblue2” module, respectively. Among them, most of the enriched terms belonged to “molecular function” and “biological process”, including “monosaccharide transmembrane transporter activity” and “sucrose transport” (Additional file 17: Table S8). *Psby* (KME = 0.993), encoding a protein in photosystem II, is one of the hub genes in the “lightpink1” module. In photosystem II, *Psby* is in close contact with *Cytb559*, which can protect photosystem II from photoinhibition for photosynthesis and provide more material and energy for cell proliferation and expansion of the silique pericarp [26]. *BnaC09g09210D* (KME = 0.982), another hub gene of the “lightpink1” module, is the homologous gene of *AtKNAT7*. In *Arabidopsis thaliana*, *AtKNAT7* is a homologous domain transcription factor of the *TALE* gene family, which is involved in the regulation of secondary cell wall biosynthesis, and its expression is up-regulated by *SND1* and *MYB46* [27]. Genes with weight values between 0.8 and 1 were screened to construct a partial co-expression network around hub genes. In the network, multiple genes were involved in cell elongation and expansion, such as *DFL2*, *CSLD3*, *TCH4*, and *CESA6* (Fig. 4d). These results suggest that photosynthesis and secondary cell wall biosynthesis may play important roles in the determination of silique length during development.

### Lignin biosynthesis plays an important role in silique elongation

The important candidate gene *BnaA9.NST2*, *BnaA7.MYB63*, *BnaA7.CCR2*, and *BnaA7.CCOAMT*, all of which are lignin biosynthesis related genes, were found in the major QTL regions of A07 and A09. Meanwhile, the pathway of “phenylpropanoid biosynthesis” was significantly enriched in different developmental stages of long silique and short silique (Additional file 18: Fig. S10a–c). These results indicate that lignin may play an important role in the development of siliques. We compared the expression levels of genes related to the lignin biosynthesis pathway in different developmental stages of long silique and short silique. Results indicate that the expression levels of lignin biosynthesis-related genes in different stages of long and short silique development were significantly different, especially in the T2 stage, when the length difference between long and short siliques increased sharply. Lignin biosynthesis related genes were highly expressed in short siliques (Additional file 18: Fig. S10d).

**Fig. 5:**
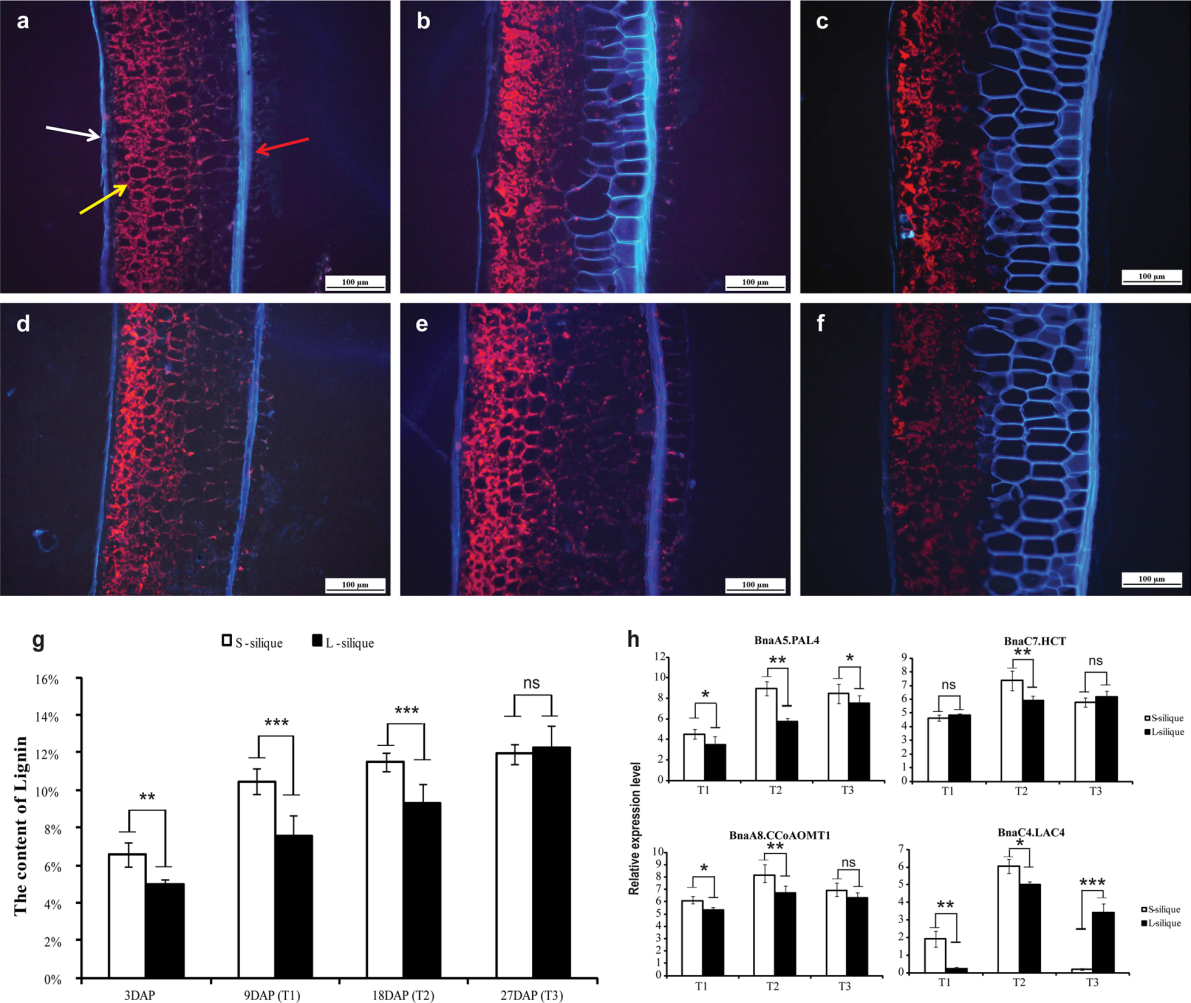
Evaluation of the phenotypic contribution of lignin to silique length. **a**–**f** Microstructure observation of the outer, middle and inner pericarp of the short and long silique. White arrows point to the outer pericarp, yellow arrows point to the middle pericarp, and red arrows point to the inner pericarp. a and d represent microscopic observations of short and long siliques at the T1 stage; **b** and **e** represent microscopic observations of short and long siliques at the T2 stage; **c** and **f** represent microscopic observations of short and long siliques at the T3 stage. Bar = 100 μm. **g** Determination of total lignin content in the silique wall of the short and long silique. **h** qRT–PCR verification of DEGs of key enzymes in the phenylpropanoid–lignin pathway in the short and long siliques at the T1, T2, and T3 stages. Statistically significant differences were revealed using a Student’s *t* test: ns ≥ 0.05, *p < 0.05, **p < 0.01, ***p < 0.001

Similar results were observed of the silique pericarp tissue section and the determination of lignin content. The lignification degree increased gradually with the silique developmental process. Especially at the T2 stage, the lignification degree of the short silique pericarp was significantly higher than that of the long silique pericarp (Fig. 5a–f). Correspondingly, the lignin content of the short silique was significantly higher than that of the long silique at the T1 and T2 stages, especially T2. However, there was no significant difference in lignin content between long and short siliques at the T3 stage (Fig. 5g). Consistent with the RNA-seq results, the expression levels of the four genes in the long silique were significantly lower than those in the short silique, especially the T2 stage (Fig. 5h). All these results suggest that lignin plays an important role in the formation of long and short siliques, especially during the rapid elongation period of the silique.

## Discussion

Population stratification is a common source of false positives in GWAS. When the population is stratified, the conventional method is to use a fixed effect covariate matrix (*Q* matrix) or PCA to control the false positive caused by population structure. Meta-analysis can increase power, reduce false-positive findings, and even identify new genetic loci, so it can solve the shortcomings of single-population GWAS [28]. In this study, we tried to perform independent GWAS by subgroups and then perform meta-analysis on the significant SNPs obtained by independent analysis. The results show that the meta-analysis confirmed 71 SNPs identified in the single-population GWAS, of which 59 SNPs had greater *p* values than in the single-population GWAS. It is worth noting that 14 SNPs undetected in GWAS were identified by meta-analysis, and seven SNPs identified in the single-population GWAS were unconfirmed by meta-analysis. S9_27787423 is closely interlocked with S9_27788376 and located inside *BnaA9.TMP-C*. The presence of S9_27788376 may mask the effect of S9_27787423, resulting in a small contribution to the phenotype. Meta-analysis showed that S9_27787423 was a significant locus. Similar to this are S7_15927280 and S9_27929254. In brief, using the meta-analysis, missing SNPs that were undetected by the single-population GWAS were retrieved, and false-positive SNPs that were identified by the single-population GWAS were filtered. The advantage of meta-analysis is that it can integrate the results of different research groups to increase the sample size of GWAS analysis, and integrate results across subgroups to avoid the influence of population stratification.

Silique is an important storage organ for photosynthetic products in rapeseed. Moderately increasing the length of the silique can increase the sink capacity of the silique, which is conducive to the transportation of filling materials to seeds. RNA-seq data show that photosynthesis may play an important role during silique development. An interesting phenomenon is that *CP12-2*, an important candidate gene related to carbohydrate synthesis and metabolism [29], was detected simultaneously within the main QTL confidence interval on A09 and C08. *BnaA9.CP12-2* and *BnaC8.CP12-2* had the same expression pattern, and there was no significant difference between long and short siliques in the T1 stage. In the T2/T3 stage, the expression in the long silique was significantly higher than that in the short silique, especially in the T2 stage when the difference in silique expanded sharply. In addition, KEGG enrichment analysis found that the synthesis and transport pathways of starch and sucrose were significantly enriched in both vertical and horizontal development stages. These results indicate that carbohydrate synthesis and metabolism may be involved in the regulation of silique development.

In this study, both linkage analysis and association analysis detected major QTL loci on A09. The QTL of SL on chromosome A09 has been a focus of research. Although major QTLs have been detected on A09 in many studies, there are differences in mapping results, indicating that there may be multiple genes controlling SL on A09. At present, two SL-related genes have been cloned on A09. *ARF18* was the first cloned gene. This gene regulates SL by regulating the auxin signalling pathway and can change seed weight without changing the number of seeds per silique [16]. Shi et al. cloned *BnaA9.CYP78A9* using map-based cloning [10]. This gene is widely expressed in rapeseed tissues and regulates silique development by affecting the auxin content. Its target gene *ARF10/16/17* is similar to the mechanism of *ARF18*, both are auxin negative response factors and inhibit downstream auxin-related genes. In our RNA-seq analysis, we found that the plant hormone signal transduction pathway was significantly enriched at different stages of silique development. In addition, linkage analysis and GWAS detected several candidate genes related to plant hormones, such as *BnaA7.ARF17* and *BnaA9.IAA30*. These results further suggest that plant hormones play an important role in silique development.

In previous studies, long and short siliques were often used as parental materials for gene mapping. Few studies have analysed the characteristics of long and short siliques growth rate. In this study, we found that there was a significant difference in the extension length between long and short siliques at 15 DAP, and the elongation time of short silique fruit was shorter. The results showed that the genes related to the lignin synthesis pathway were significantly differentially expressed between long and short siliques in the early, middle, and late stages of development. In addition, *BnaA7.MYB63* and *BnaA9.NST2*, the key genes of lignin synthesis, were candidate genes detected by linkage analysis and GWAS. Further determination of lignin content also showed that there was a significant difference in lignin content between long and short siliques. The results suggest that lignin may be one of the important factors determining the silique length. The differential expression of lignin biosynthesis-related genes leads to the differences in lignin content. Lignin accumulation inhibits the expansion of pericarp cells and finally affects silique length. For the same environment, the yield of short siliques rapeseed is often lower than that of long siliques rapeseed. In addition, the seed lignin content is significantly negatively correlated with the seed oil content [30]. The cost of degrading plant cell wall polysaccharides is high by enzymatic or chemical methods. In addition, lignin affects the processing and utilization of cell wall polysaccharides in many industrial and agricultural aspects. Therefore, strategically reducing lignin content may improve rapeseed yield and the efficiency of biomass utilization for bioenergy.

## Conclusions

In the present study, we identified QTLs for SL using an RIL population and two independent GWAS populations. Major QTLs on chromosomes A07, A09, and C08 were stably detected in all environments from all populations. Combining RNA-seq and WGCNA, we found that carbohydrate synthesis and metabolism and plant hormones are involved in the regulation of silique development, and the difference in lignin accumulation may also be a key factor affecting silique length.

## Materials and methods

### Plant materials and trait measurement

The genetic map that we developed earlier using a recombinant inbred line (RIL) mapping population with 172 lines was used for QTL mapping in this study [31]. A previously reported GWAS population with 520 accessions (referred to as the 60 K population) was used for association analysis of silique length [32], and another previously reported GWAS population with 588 accessions (referred to as the WGR population) was also used in this study [25]. There are 211 overlapping material between 60 K population and WGR population. The RIL population and the parents were grown in five environments: winter of 2016, 2017, and 2018 at Southwest University in Beibei, Chongqing, China (cq; 29.80° N, 106.40° E) and summer of 2018 and 2019 in Xian, Shanxi, China (xa; 34.27° N, 108.08° E). The 60 K population and WGR population were cultivated under natural growing conditions at the experimental farm of Southwest University for 2015 and 2017, respectively. All lines were arranged in a randomized complete block design with three replicates, and each line was planted in two rows of 10 plants per row, with 30 cm between rows and a distance of 20 cm between plants within each row. After the silique was mature, a ruler was used to measure the silique length (silique body length), and the silique beak length was not included in the silique length. Five siliques were randomly measured for each line of the RIL population, ten siliques were randomly measured for each accession of the GWAS population, and the average value was taken.

### QTL mapping, GWAS and meta-GWAS

QTL mapping was performed by composite interval mapping (CIM) using the software WinQTL Cartographer 2.5 software [33], and LOD thresholds for QTL detection were set to *P* = 0.05 and were determined using permutation tests with 1000 permutations.

The GWAS was conducted using the general linear model (GLM), mixed linear model (MLM) [34], compressed mixed linear model (CMLM) [34], bayesian-information and linkage-disequilibrium iteratively nested keyway (BLINK) [35], and fixed and random model circulating probability unification (FarmCPU) [36] with a total of 31,839 and 670,028 SNPs (miss data < 20%, MAF > 0.05) in the GAPIT R package [37]. For meta-GWAS, we first divided the WGR population into three subgroups (spring, winter, and semi-winters), and then conducted GWAS independently. Then, pooled data from the GWAS results of three subgroups were used for meta-analysis. The meta-analysis was performed using METAL with the *p* value, *β*-coefficients, and standard errors from single-subgroup GWAS [38]. In the single-population GWAS and meta-analysis, the genome-wide significant (1/*N*) thresholds by Bonferroni correction, in which N is the number of SNPs, were used in the analysis.

An LD block was generated using Haploview v4.2 via the four-gamete rule [39]. The parameters were set as follows: the MAF was 0.05, the maximum number of Mendel errors was 1, the Hardy–Weinberg *p* value cutoff was 0.001, and the minimum genotype was 75%.

### RNA-seq and WGCNA

Based on multi-year phenotypic observations, Z068 and Z191, from the RIL population, were used as representatives of the S- and L-siliques. Silique pericarps of five lines with S- and L-siliques were collected 9, 18, and 27 DAP with two replications, and the samples were immediately frozen in liquid nitrogen and stored at − 80 °C for RNA-Seq. ST1, ST2, and ST3 represent S-silique at 9, 18 and 27 DAP, respectively; LT1, LT2, and LT3 represent L-silique at 9, 18 and 27 DAP, respectively. The libraries were sequenced on the Illumina HiSeq 4000 platform. After the removal of low-quality reads, the clean reads were aligned to the *B. napus* reference genome. Fragments per kilobase million (FPKM) values were calculated to estimate gene expression levels. Differentially expressed genes (DEGs) were identified in each pair of samples using the criteria of an false discovery rate (FDR) < 0.01 and |log2(fold change)| ≥ 1.5. The genes with FPKM values in more than 12 samples lower than 0.3 were filtered out.

WGCNA was performed using averaged FPKM values and the WGCNA package in R software, the analysis method based on tutorial of WGCNA official website (https://labs.genetics.ucla.edu/horvath/CoexpressionNetwork/Rpackages/WGCNA/) [40]. A total of 5809 non-redundant DEGs (RNA-seq) and putative candidate genes (QTL mapping and GWAS results) were included in the WGCNA workflow. Briefly, cluster analysis was first carried out to remove outliers, and the scale-free topology criterion was used to determine the soft threshold, which is defined as the similarity relationships between gene-pairs and is obtained by computing the unsigned Pearson’s correlation matrix, and threshold parameter beta selects the value at which the fitting curve first approaches 0.9. Subsequently, the network was constructed using a step-by-step method by turning the adjacency matrix into a topological overlap matrix (TOM) and calling the hierarchical clustering function, and network modules were identified using a dynamic tree cut algorithm with a minimum cluster size of 30 and merging the threshold function at 0.25. To identify hub genes within the modules, the module membership (MM) for each gene was calculated based on the Pearson correlation between the expression level and the module eigengene. Gene ontology (GO) enrichment analysis and Kyoto Encyclopedia of Genes and Genomes (KEGG) were performed using the OmicShare tools, and network depictions were visualized using Cytoscape v3.2.1.

### Candidate genes analysis and qRT–PCR

The putative candidate genes were searched within the interval of the associated SNPs (± 200 kb) based on the Darmor-bzh *B. napus* reference genome v4.1. Functional annotation was implemented to predict the function of candidate genes using a Blastp program against to the *Arabidopsis thaliana* TAIR10 protein database.

The expression levels of silique length-related genes were validated by real-time PCR (qRT–PCR) using a CFX96 Real-time System (BIO-RAD, USA). In brief, 1 µg of RNA from each sample was used for cDNA synthesis; the expression of the silique length-related genes in different rapeseed samples was evaluated using SYBR® Premix (TIANGEN, Beijing, China) and a CFX96 Real-time System (BIO-RAD, USA). Gene-specific primers are listed in Additional file 19: Table S9. Average Cq values were calculated from three replicates, and expression levels were normalized to the reference gene *Bna.Actin7* using the 2^−ΔΔCt^ method.

### Microscopic observation and lignin quantification

Using OCT embedding tissue freezing slice technique, the microstructure of the fresh pericarp of 9, 18, and 27 DAP from extreme lines were observed. After successful embedding, all samples were cut continuously by the ice cutter with 30 μm. The sections with the intact structure were placed under the Nikon ECLIPSE E600 microscope, observed under ultraviolet light with 10× and 20× objective lenses, and photographed and recorded.

The acetyl bromide method is used to determine lignin content. Briefly, all pericarps were dried to constant weight in an oven at 70 °C, and the samples were ground to pass an 80-mesh screen in the microball mill prior to drying over P2O5 in a vacuum desiccator. The cell wall was isolated by four stage Soxhlet extraction. The dried cell wall samples about 2 mg were added to a 2 mL glass tube with 200 μL of 25% acetyl bromide in acetic acid. The tubes were tightly sealed and put in a 50 °C water bath for 2 h with shaking at 30 min intervals. After cooling the tubes to room temperature, the samples were transferred to 15 mL stoppered graduated centrifuge tube containing 800 μL 2 M NaOH and 140 μL 0.5 M hydroxylamine hydrochloride. Then acetic acid is used to rinse tubes and made up to 15 mL. The absorbance of the solutions was read at 280 nm using a Varian Cary 50 spectrophotometer. A blank was included to correct for background absorbance by the reagents. The lignin content was estimated according to the following equation: %ABSL = (abs/(Coeff × 1 cm))  *  ((15 mL * 100%)/weight(mg)), where abs represents absorbance, Coeff represents extinction coefficient [41].

### Statistical analysis

Analysis of variance (ANOVA) of phenotypic data was conducted using “aov” function of R. The broad-sense heritability was estimated according to the following equation: *h*^2^ = б^2^g/(б^2^g + б^2^ge/*n* + б^2^e/*nr*) × 100%, where б^2^g represents the genetic variance, б^2^ge represents the interaction variance between genotypes and environments, б^2^e represents the error variance, *n* represents the number of environments, and *r* represents the number of replicates within each environment, respectively [42]. To make the results more accurate, the lme4 package in R was used to estimate best linear unbiased predictions (BLUPs) and best linear unbiased estimates (BLUEs) across multi environment on a per line basis for silique length [43]. The BLUP and BLUE values were used as trait values for QTL analysis.

## Supplementary Information

**Additional file 1: Table S1.** Phenotypic variations in silique length (SL) of the *B. napus* accessions.

**Additional file 2: Figure S1.** Distribution of silique length in the RIL and WGAS populations. (a) Representative RIL population. 16SL-cq, 17SL-cq, and 18SL-cq represent silique length from Chongqing in 2016, 2017, and 2018, respectively; 18SL-xa and 19SL-xa represent silique length from Xi’an in 2018 and 2019, respectively; BLUP and BLUE represent best linear unbiased predictions and best linear unbiased estimates, respectively. (b) Representative GWAS population.

**Additional file 3: Figure S2.** Pearson’s correlation coefficients of silique length and yield-related traits. The upper triangle shows statistics (correlation coefficient), the lower triangle shows smooth spline regression, and diagonals are histograms. SL, silique length; TSW, thousand seed weight; SN, seed number per silique; YP, yield per plant; SNPP, seed number per plant; SMI, siliques per main inflorescence; HI, harvest index; SOC, seed oil content.

**Additional file 4: Table S2.** Quality of sequencing data.

**Additional file 5: Table S3.** Statistical table of sequence alignment results between sample sequencing data and selected reference genome.

**Additional file 6: Figure S3.** Overview of RNA-seq. (a) Gene expression profile of each sample. (b) Comparison diagram of the FPKM density distribution of each sample. (c) Correlation heatmap of 12 samples. (d) Venn diagram of DEGs. (e) The number of up- and down-regulated DEGs.

**Additional file 7: Table S4.** The number of differentially expressed genes (DEGs).

**Additional file 8: Figure S4.** Top 20 KEGG enriched pathways in set of T1 vs. T2, T1 vs. T3, and T2 vs. T3 between short silique and long silique.

**Additional file 9: Table S5.** QTL for silique length detected in the RILs across five environments, BLUE, and BLUP.

**Additional file 10: Figure S5.** Genetic linkage map and QTL detection of the silique length in the RIL population. For simplicity, only the markers in the QTL confidence intervals, along with the terminal two markers at each end of the QTL-containing chromosomes, are shown.

**Additional file 11: Figure S6.** Quantile–quantile plots of estimated − log10(*P*) from association analysis of SL. (a) Quantile–quantile plots of the 60 K population; (b) quantile–quantile plots of the WGR population.

**Additional file 12: Table S6.** Genome-wide significant association signals of SL using the single-population GWAS and meta-GWAS.

**Additional file 13: Table S7.** Candidate genes within the linked genomic region of single nucleotide polymorphisms (SNPs) most highly associated with SL in *B. napus*.

**Additional file 14: Figure S7.** Box plot of genotype frequency distribution of different SNP loci and polymorphisms in the candidate region. (a) S9_27782829 genotype frequency distribution. (b) S9_27788376 genotype frequency distribution. ** represents significance *p* < 0.01. (c) Genomic diversity of landrace (the red line) and pseudo-wild ancestral (the blue line) rapeseed on C08, respectively.

**Additional file 15: Figure S8.** Mapping of QTL on A07 using the approach of linkage analysis and association analysis. The Manhattan plots of chromosome A07 are plotted in the top left graph, and the green line indicates the threshold level log(1/*N*) = 5.58. The QTL on A07 detected in the RIL population is shown in the top right graph. The diagram below these two graphs shows the location of the reference genome region on A07 corresponding to the QTL and LD block analysis of this region. The red gene ID represents that the gene is an important candidate gene.

**Additional file 16: Figure S9.** Cluster analysis of the top twenty genes of KME values in modules. (a) Lightpink1 module. (b) Chocolate3 module. (c) Darkgoldnrod4 module. (d) Lightblue2 module.

**Additional file 17: Table S8.** GO enrichments of modules.

**Additional file 18: Figure S10.** Top 20 KEGG enriched pathways in the set of ST1 vs. LT1, ST2 vs. LT2, and ST3 vs. LT3 and the expression analysis of DEGs of key enzymes in the phenylpropanoid–lignin pathway. (a) ST1 vs. LT1. (b) ST2 vs. LT2. (c) ST3 vs. LT3. (d) The expression analysis of DEGs of key enzymes in phenylpropanoid–lignin pathway. *PAL*, phenylalanine ammonia-lyase; *C4H*, cinnamate 4-hydroxylase; *4CL*, 4-coumarate acid: CoA ligase; *HCT*, hydroxycinnamoyl-CoA shikimate/quinate transferase; *C3H*, coumarate 3-hydroxylase; *CCoACOMT*, caffeoyl-CoA-*O*-methyltransferase; *CCR*, cinnamoyl CoA reductase; *F5H*, ferulate 5-hydroxylase; *COMT*, caffeic acid *O*-methyltransferase; *CAD*, cinnamyl alcohol dehydrogenase; *PER*, peroxidase; *LAC*, laccase.

**Additional file 19: Table S9.** Primers used for qRT–PCR.

## Data Availability

The data sets supporting the conclusions of this article are included within the article and its additional files, and the raw RNA-Seq data have been deposited in the NCBI database under BioProject accession code PRJNA752824.
