# Supplementary material for: Genome-wide association study and transcriptome analysis dissect the genetic control of silique length in Brassica napus L
Source: Biotechnol Biofuels. 2021 Nov 7;14:214. doi: 10.1186/s13068-021-02064-z (PMC8573943; doi:10.1186/s13068-021-02064-z)
Supplement: Supplementary file 1 — Additional file 1: Table S1. Phenotypic variations in silique length (SL) of the B. napus accessions. [file 13068_2021_2064_MOESM1_ESM.docx]

**Additional file 1: Table S1 Phenotypic variations in silique length (SL) the *B. napus* accessions.**

| **population** | **Traits** | RIL P1 | RIL P2 | **Minimum** | **Maximum** | **Mean+SD** | **CV%^a^** | **Skewness** | **Kurtosis** | **G** | **E** | **G*E** | ***h2*^b^** |
| --- | --- | --- | --- | --- | --- | --- | --- | --- | --- | --- | --- | --- | --- |
| RIL | 16SL-cq | 5.81 | 8.26 | 4.82 | 9.65 | 7.28+1.36 | 18.74 | -0.25 | -1.11 | ****** | ****** | ****** | 76.26% |
|  | 17SL-cq | 5.14 | 7.67 | 4.43 | 8.60 | 6.53+1.15 | 17.57 | -0.30 | -1.09 |  |  |  |  |
|  | 18SL-cq | 5.85 | 8.42 | 5.16 | 10.51 | 7.85+1.51 | 19.19 | -0.29 | -1.02 |  |  |  |  |
|  | 18SL-xa | 5.73 | 8.15 | 5.29 | 9.75 | 7.51+1.22 | 16.22 | -0.28 | -1.09 |  |  |  |  |
|  | 19SL-xa | 5.45 | 8.53 | 4.81 | 10.89 | 7.38+1.44 | 19.58 | 0.16 | -0.40 |  |  |  |  |
| GWAS-60K | 60K(2014) |  |  | 3.52 | 9.90 | 5.75+0.91 | 15.78 | 0.92 | 1.79 |  |  |  |  |
| GWAS-WGR | WGR(2019) |  |  | 3.39 | 12.74 | 5.95+1.27 | 21.36 | 1.27 | 3.27 |  |  |  |  |
|  | Winter subgroup |  |  | 3.41 | 12.74 | 5.51+1.45 | 26.36 | 3.18 | 11.42 |  |  |  |  |
|  | Spring subgroup |  |  | 3.46 | 10.52 | 5.31+1.12 | 21.11 | 2.27 | 7.87 |  |  |  |  |
|  | Semi-winter subgroup |  |  | 3.39 | 11.68 | 6.07+1.23 | 20.21 | 1.11 | 2.23 |  |  |  |  |

** The values are significant at P < 0.01 for the effect of genotype (G), environment (E) and genotype by environment interaction (G×E) on phenotypic variance estimated by two-way ANOVA.

^a^ CV is an abbreviation of coefficient of variation, which was estimated as the ratio of the standard deviation to the mean of all accessions.

^b^ *h^2^* is broad-sense heritability; *h^2^* = б^2^_g_/(б^2^_g_+б^2^_ge_/n+б^2^_e_/nr)×100%, where б^2^_g_ is the genetic variance, б^2^_ge_ is the variance due to the G × E interaction, б^2^_e_ represents the residual error, n is the number of environments (years), and r is number of replicates.
