# Supplementary figures and images for: Genome-wide association study and transcriptome analysis dissect the genetic control of silique length in Brassica napus L
Source: Biotechnol Biofuels. 2021 Nov 7;14:214. doi: 10.1186/s13068-021-02064-z (PMC8573943; doi:10.1186/s13068-021-02064-z)

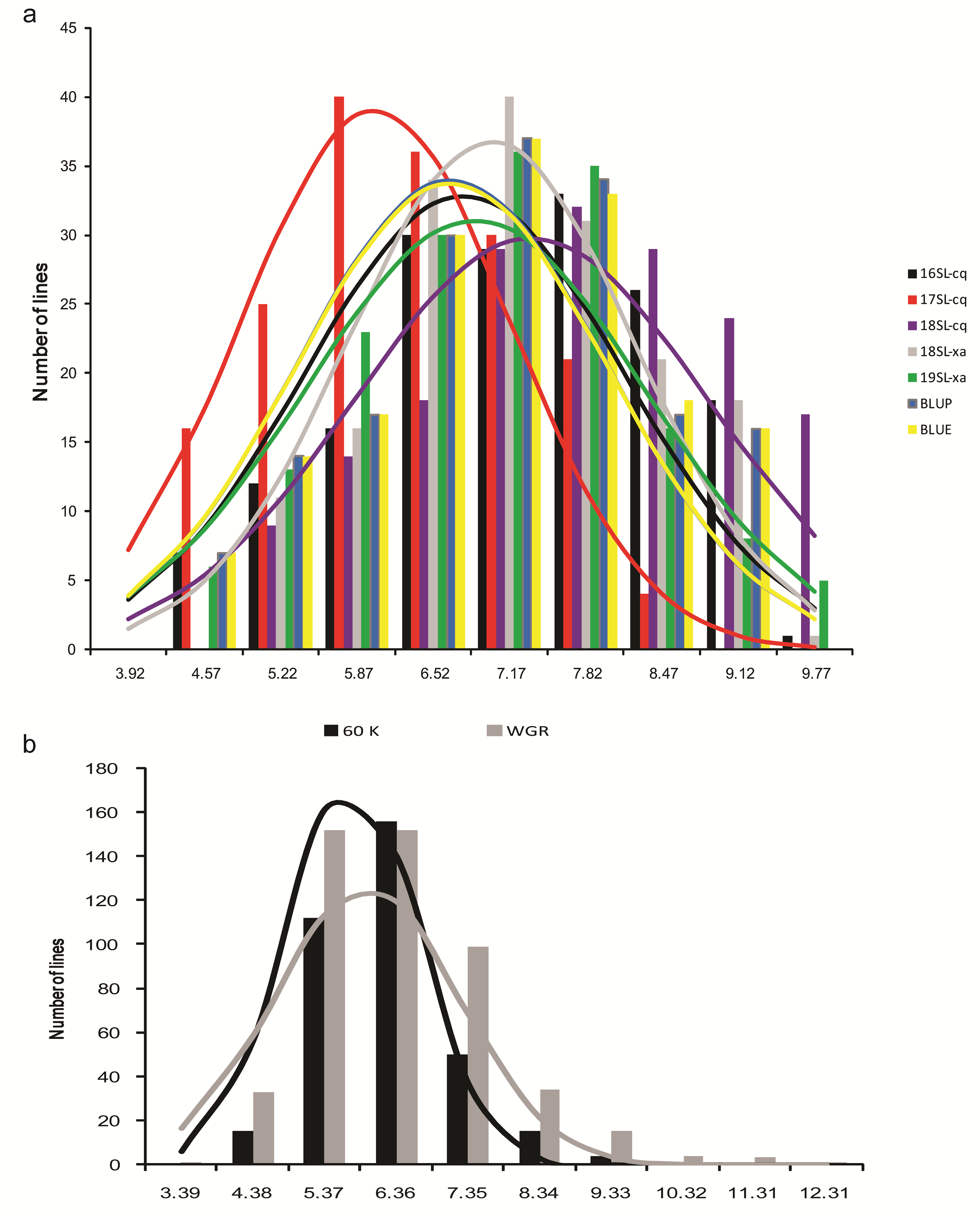

Supplement: Supplementary file 2 — Additional file 2: Figure S1. Distribution of silique length in the RIL and WGAS populations. (a) Representative RIL population. 16SL-cq, 17SL-cq, and 18SL-cq represent silique length from Chongqing in 2016, 2017, and 2018, respectively; 18SL-xa and 19SL-xa represent silique length from Xi’an in 2018 and 2019, respectively; BLUP and BLUE represent best linear unbiased predictions and best linear unbiased estimates, respectively. (b) Representative GWAS population. [file 13068_2021_2064_MOESM2_ESM.tif]

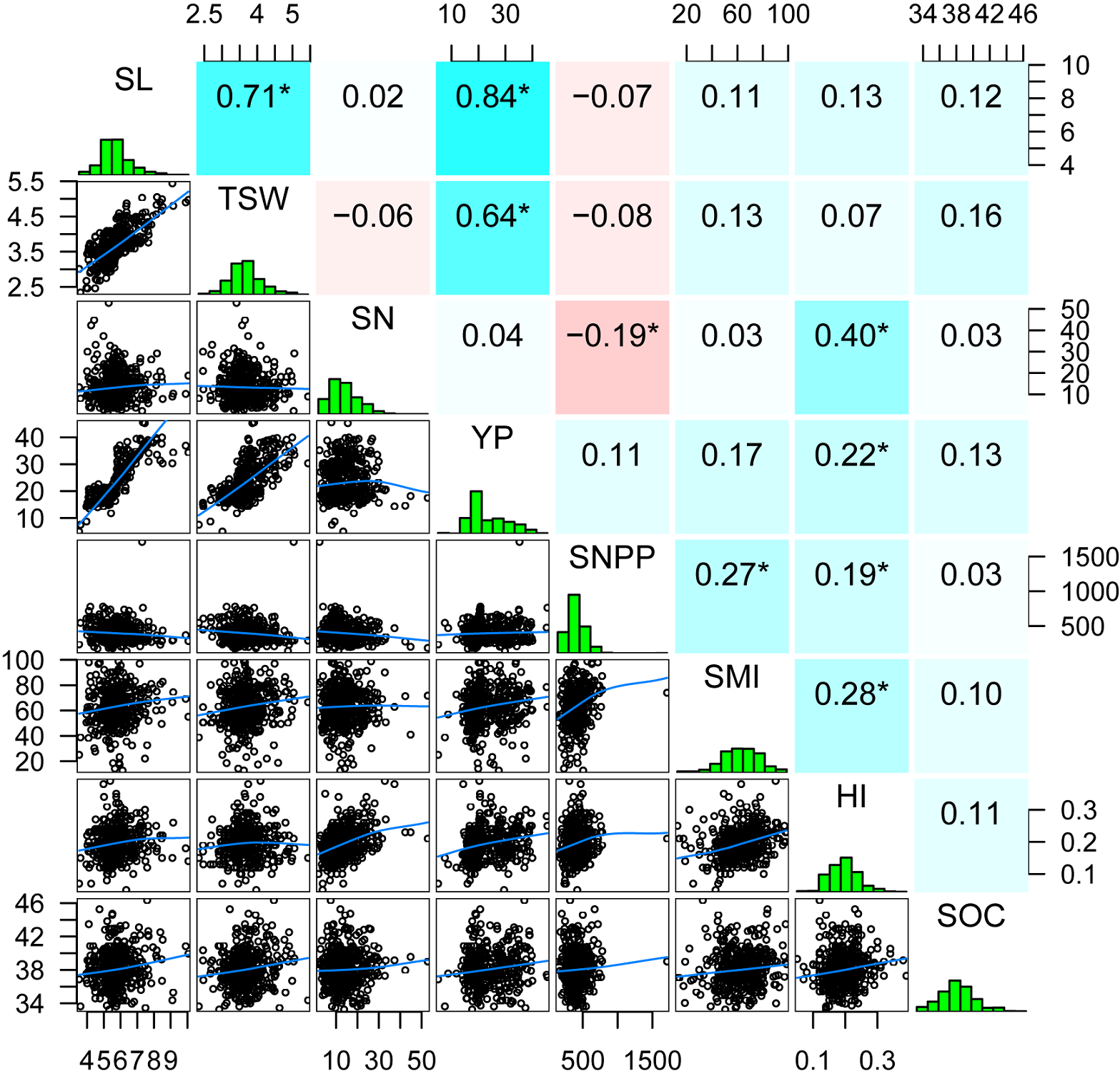

Supplement: Supplementary file 3 — Additional file 3: Figure S2. Pearson’s correlation coefficients of silique length and yield-related traits. The upper triangle shows statistics (correlation coefficient), the lower triangle shows smooth spline regression, and diagonals are histograms. SL, silique length; TSW, thousand seed weight; SN, seed number per silique; YP, yield per plant; SNPP, seed number per plant; SMI, siliques per main inflorescence; HI, harvest index; SOC, seed oil content. [file 13068_2021_2064_MOESM3_ESM.tif]

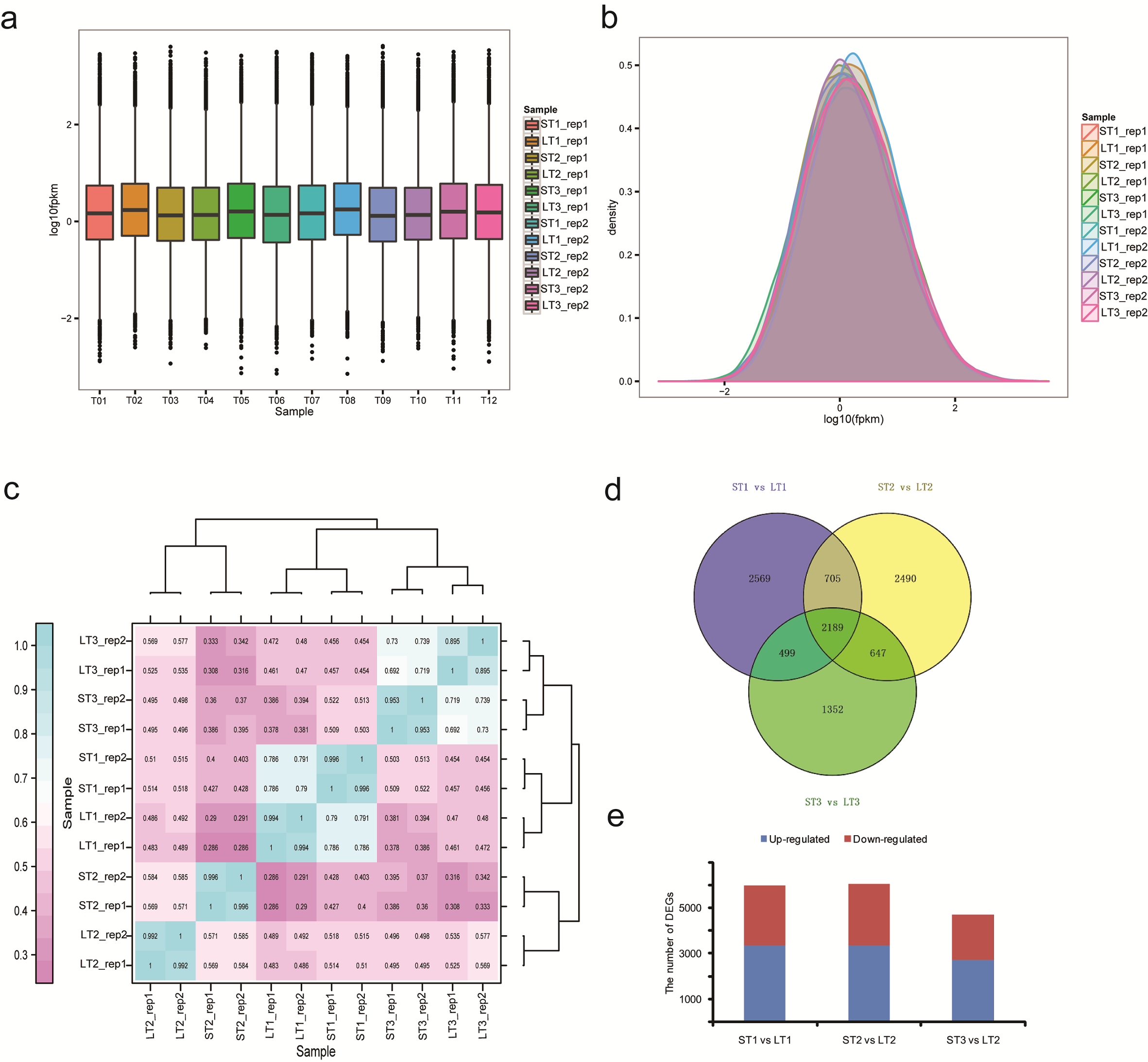

Supplement: Supplementary file 6 — Additional file 6: Figure S3. Overview of RNA-seq. (a) Gene expression profile of each sample. (b) Comparison diagram of the FPKM density distribution of each sample. (c) Correlation heatmap of 12 samples. (d) Venn diagram of DEGs. (e) The number of up- and down-regulated DEGs. [file 13068_2021_2064_MOESM6_ESM.tif]

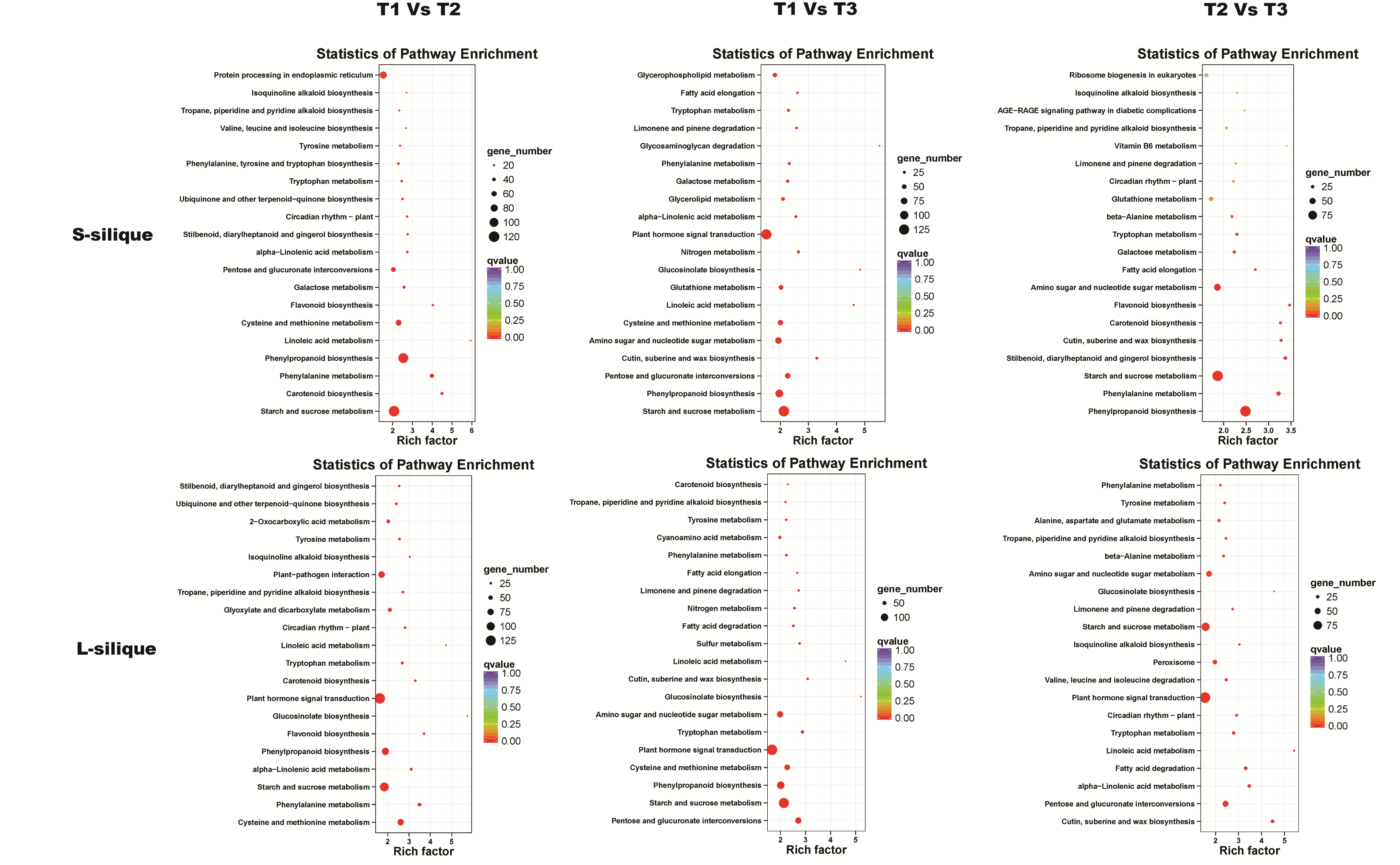

Supplement: Supplementary file 8 — Additional file 8: Figure S4. Top 20 KEGG enriched pathways in set of T1 vs. T2, T1 vs. T3, and T2 vs. T3 between short silique and long silique. [file 13068_2021_2064_MOESM8_ESM.tif]

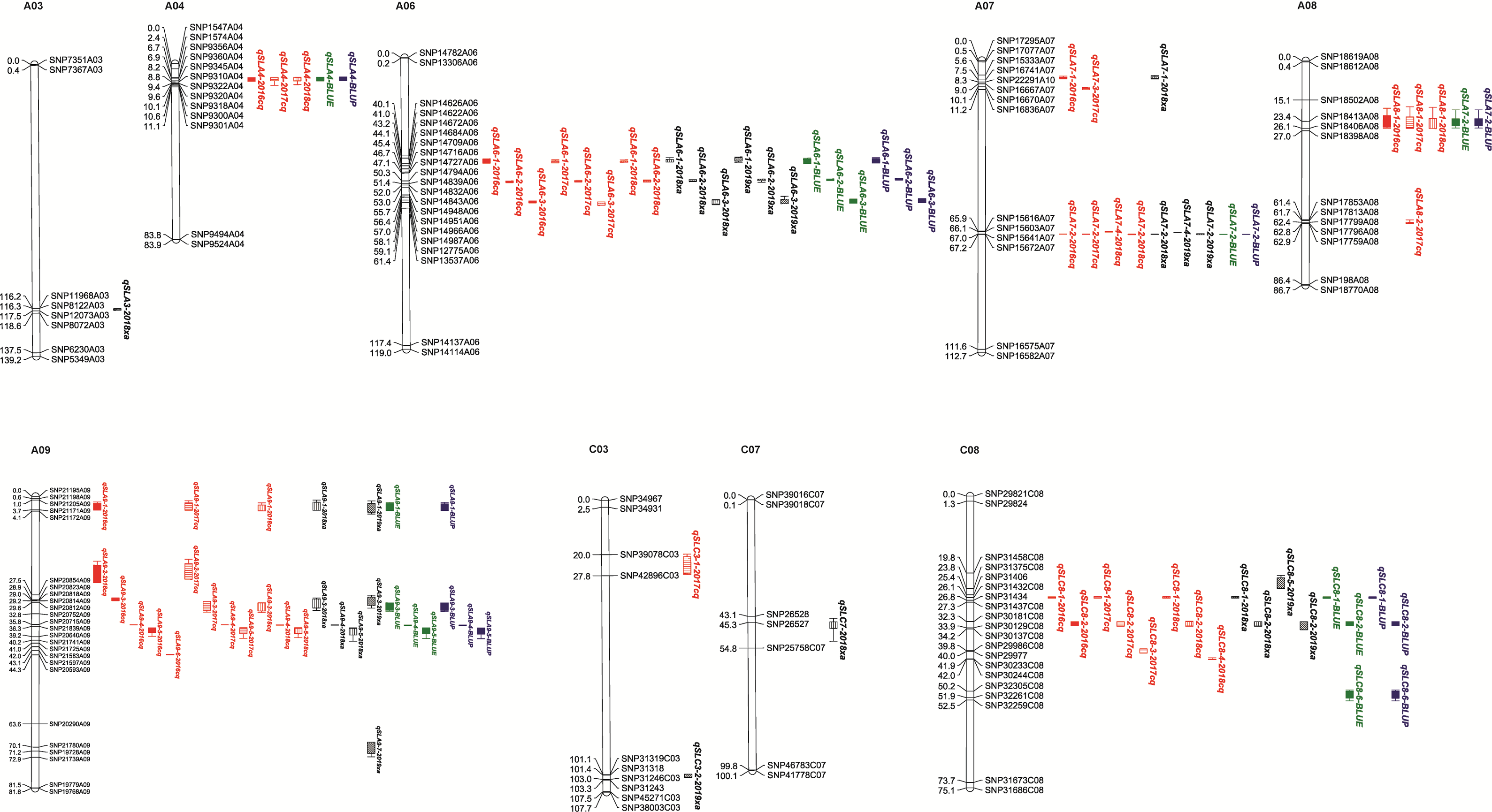

Supplement: Supplementary file 10 — Additional file 10: Figure S5. Genetic linkage map and QTL detection of the silique length in the RIL population. For simplicity, only the markers in the QTL confidence intervals, along with the terminal two markers at each end of the QTL-containing chromosomes, are shown. [file 13068_2021_2064_MOESM10_ESM.tif]

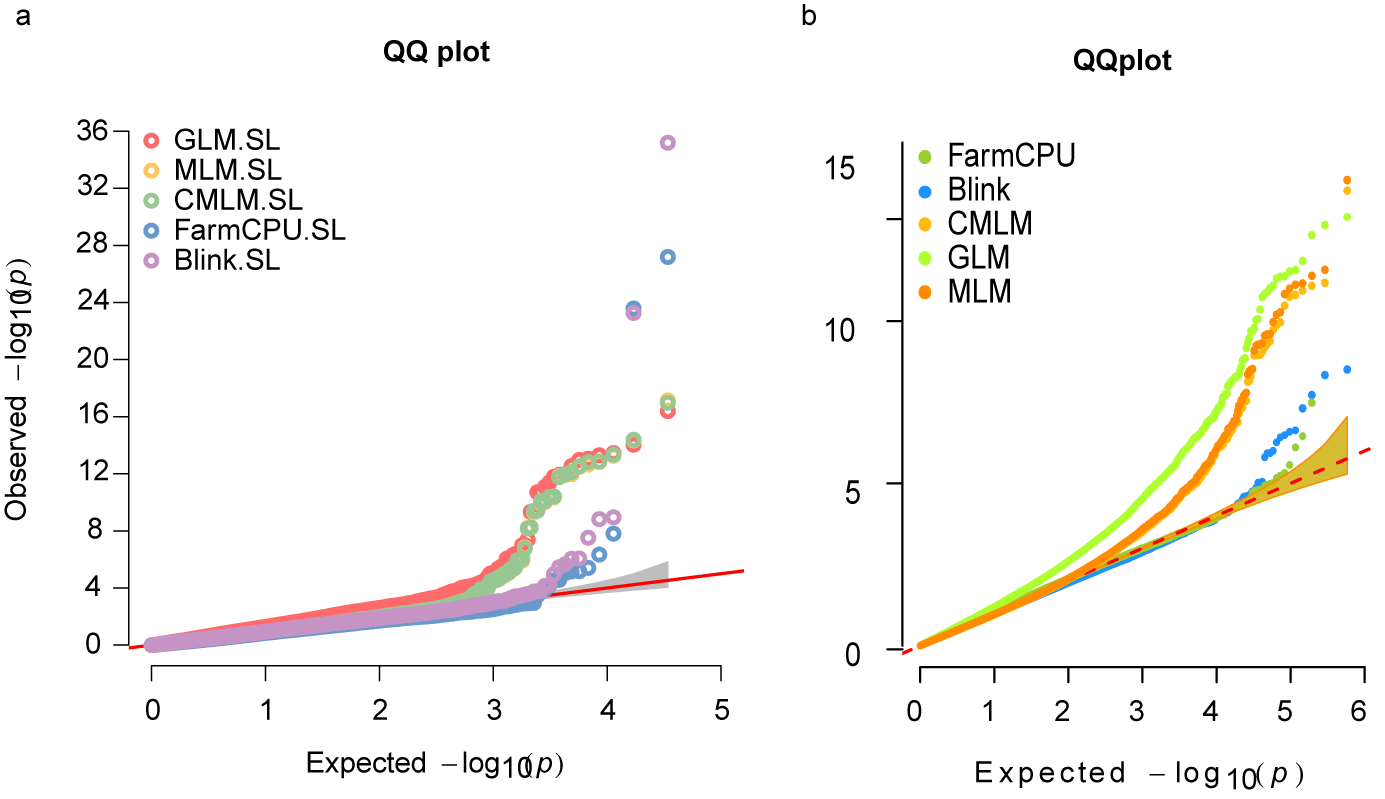

Supplement: Supplementary file 11 — Additional file 11: Figure S6. Quantile–quantile plots of estimated − log10(P) from association analysis of SL. (a) Quantile–quantile plots of the 60 K population; (b) quantile–quantile plots of the WGR population. [file 13068_2021_2064_MOESM11_ESM.tif]

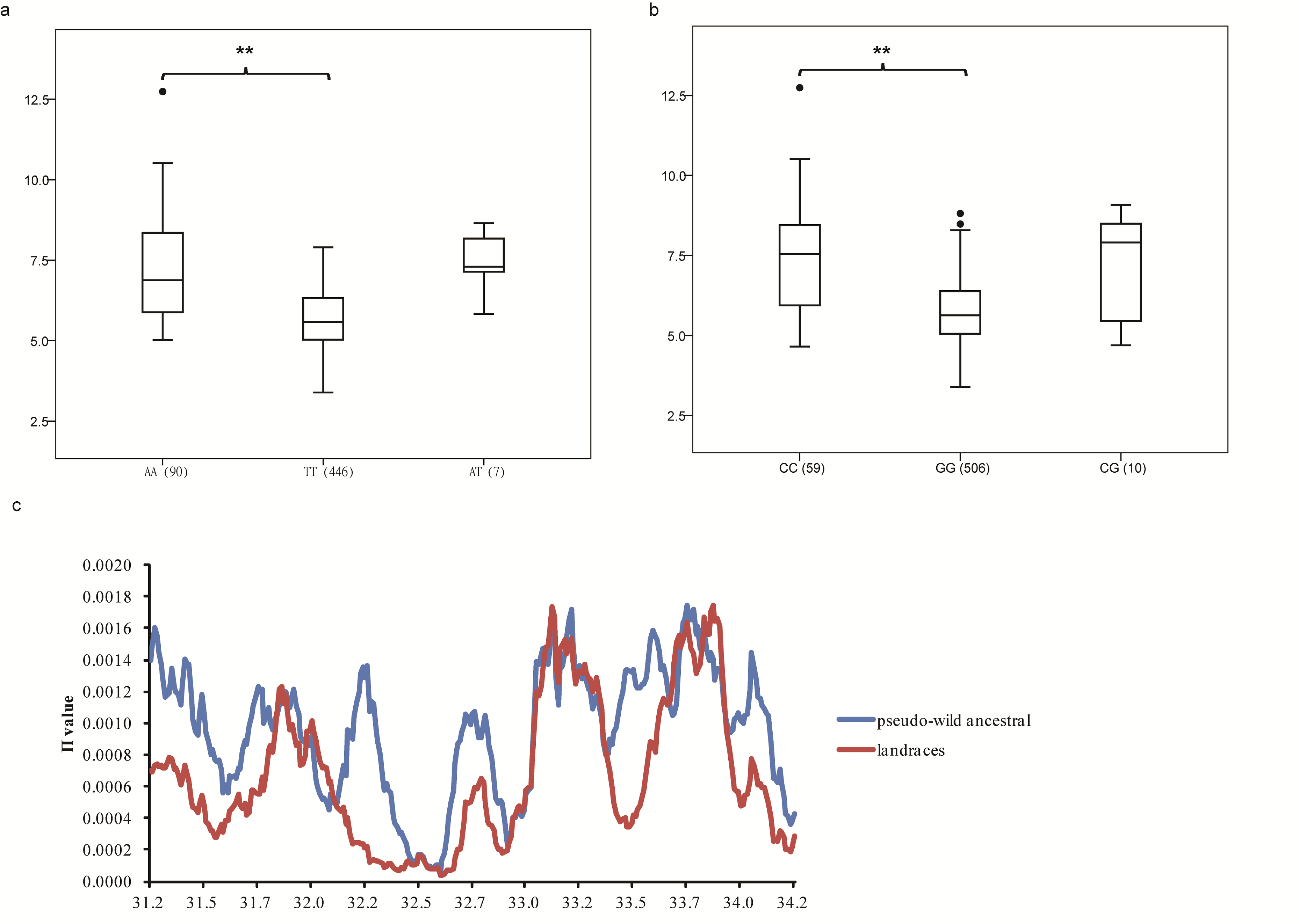

Supplement: Supplementary file 14 — Additional file 14: Figure S7. Box plot of genotype frequency distribution of different SNP loci and polymorphisms in the candidate region. (a) S9_27782829 genotype frequency distribution. (b) S9_27788376 genotype frequency distribution. ** represents significance p < 0.01. (c) Genomic diversity of landrace (the red line) and pseudo-wild ancestral (the blue line) rapeseed on C08, respectively. [file 13068_2021_2064_MOESM14_ESM.tif]

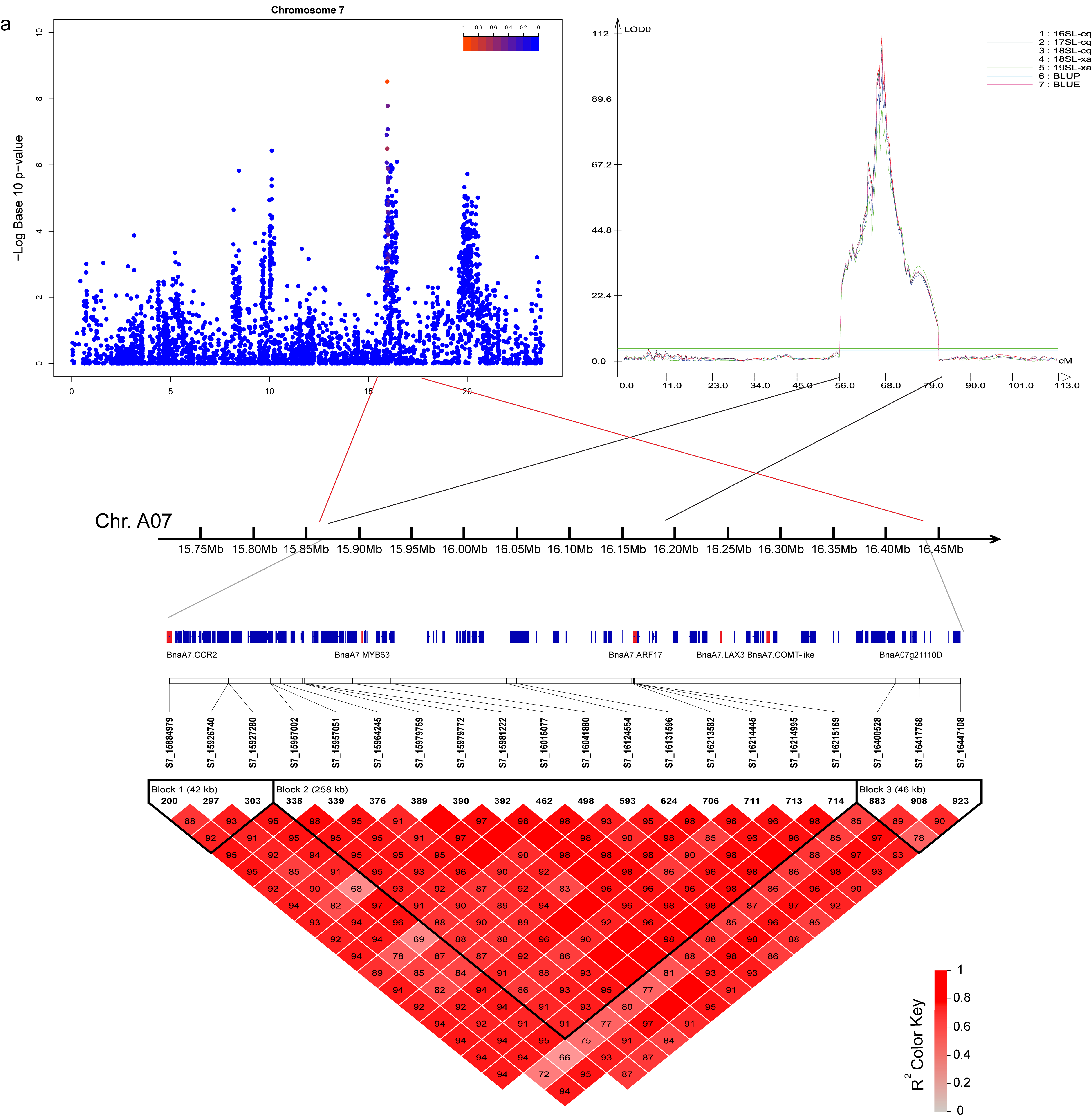

Supplement: Supplementary file 15 — Additional file 15: Figure S8. Mapping of QTL on A07 using the approach of linkage analysis and association analysis. The Manhattan plots of chromosome A07 are plotted in the top left graph, and the green line indicates the threshold level log(1/N) = 5.58. The QTL on A07 detected in the RIL population is shown in the top right graph. The diagram below these two graphs shows the location of the reference genome region on A07 corresponding to the QTL and LD block analysis of this region. The red gene ID represents that the gene is an important candidate gene. [file 13068_2021_2064_MOESM15_ESM.tif]

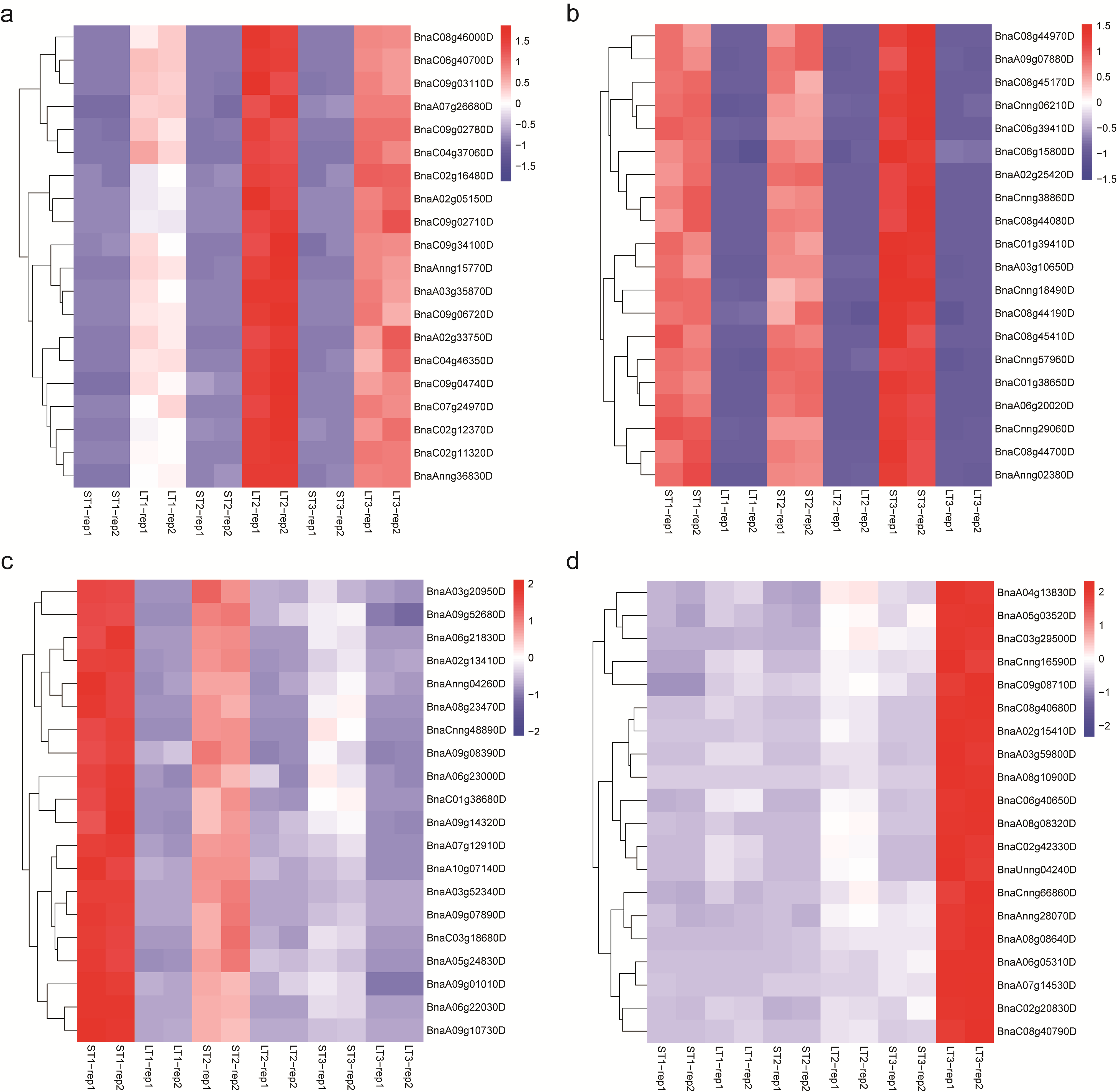

Supplement: Supplementary file 16 — Additional file 16: Figure S9. Cluster analysis of the top twenty genes of KME values in modules. (a) Lightpink1 module. (b) Chocolate3 module. (c) Darkgoldnrod4 module. (d) Lightblue2 module. [file 13068_2021_2064_MOESM16_ESM.tif]

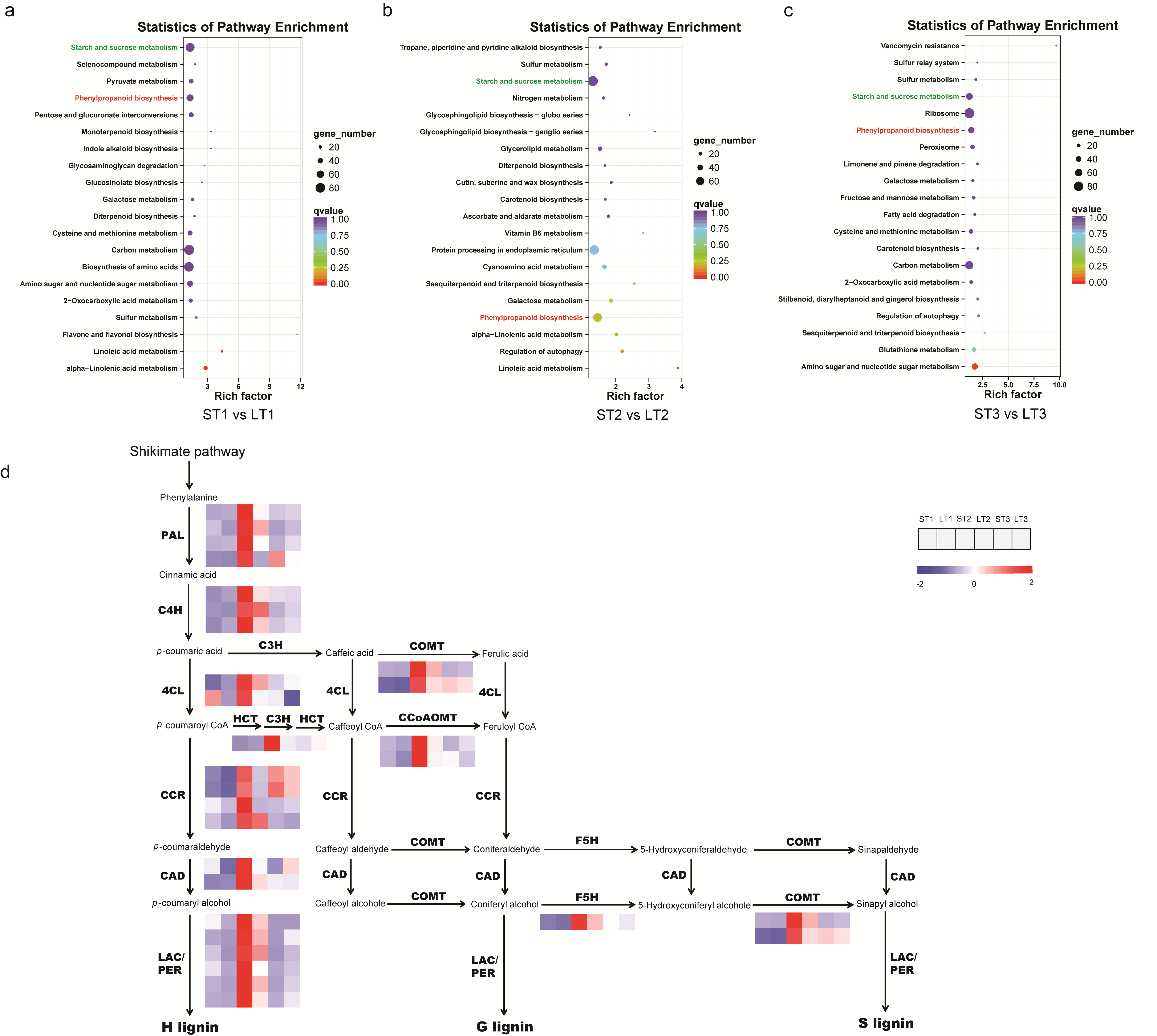

Supplement: Supplementary file 18 — Additional file 18: Figure S10. Top 20 KEGG enriched pathways in the set of ST1 vs. LT1, ST2 vs. LT2, and ST3 vs. LT3 and the expression analysis of DEGs of key enzymes in the phenylpropanoid–lignin pathway. (a) ST1 vs. LT1. (b) ST2 vs. LT2. (c) ST3 vs. LT3. (d) The expression analysis of DEGs of key enzymes in phenylpropanoid–lignin pathway. PAL, phenylalanine ammonia-lyase; C4H, cinnamate 4-hydroxylase; 4CL, 4-coumarate acid: CoA ligase; HCT, hydroxycinnamoyl-CoA shikimate/quinate transferase; C3H, coumarate 3-hydroxylase; CCoACOMT, caffeoyl-CoA-O-methyltransferase; CCR, cinnamoyl CoA reductase; F5H, ferulate 5-hydroxylase; COMT, caffeic acid O-methyltransferase; CAD, cinnamyl alcohol dehydrogenase; PER, peroxidase; LAC, laccase. [file 13068_2021_2064_MOESM18_ESM.tif]
